# Structure-based molecular modeling of a novel S-Remiketamine scaffold and derived arylcyclohexanone analogues: NMDA receptor dynamics, MM-GBSA ranking, and CES1-Oriented structural compatibility

**DOI:** 10.3389/fchem.2026.1922077

**Published:** 2026-09-03

**Authors:** Ahmed A. A. M. M. Alkhatip, Usama Raza, Ehab Farag, Mohamed Khaled Hamza, Hisham Hosny, Amr Sallam, Ahmed M. G. Farag, Mohamed Wagih, Amr Naguib, Ahmed Mostafa Abdulmegid, Haytham Algameel, Mahmoud Hussein Bahr

**Affiliations:** 1 Department of Anaesthesia, Birmingham Children’s Hospital, Birmingham, United Kingdom; 2 Department of Anesthesia, Pain Management and Surgical Intensive Care, Beni-Suef University Hospital and Faculty of Medicine, Beni Suef University, Beni Suef, Egypt; 3 Department of Pharmaceutical Chemistry, Dow College of Pharmacy, Dow University of Health Sciences, Karachi, Pakistan; 4 Department of Anesthesia, Kasr Al Ainy Faculty of Medicine and Cairo University Hospitals, Cairo University, Cairo, Egypt; 5 Department of Cardiothoracic Anaesthesia and Intensive Care Medicine, Freeman Hospital, Newcastle upon Tyne Hospitals NHS Foundation Trust, Newcastle upon Tyne, United Kingdom; 6 Department of Anaesthesia, St. Vincent’s University Hospital, Dublin, Ireland; 7 Department of Cardiac Anaesthesia, King Abdullah Medical City, Makkah, Saudi Arabia; 8 Department of Anaesthesia, Aberdeen Royal Infirmary, Aberdeen, United Kingdom

**Keywords:** carboxylesterase 1, computer-aided drug design, MM-GBSA, molecular dynamics, molecular modeling, NMDA receptor, S-ketamine, S-Remiketamine

## Abstract

**Introduction:**

Short-acting ketamine-related scaffold design remains an important medicinal-chemistry problem, but computational studies require conservative interpretation until receptor, enzymatic, and pharmacokinetic validation is available.

**Methods:**

S-ketamine, S-Remiketamine, and R1-R20 analogues were assessed using ligand curation, NMDA receptor docking, cross-target docking, explicit-solvent molecular dynamics, MM-GBSA analysis, CES1-oriented geometric assessment, and predictive ADMET/metabolite-route analysis.

**Results:**

Static NMDA docking ranked R2 highest (Glide score -7.308 kcal/mol), while S-Remiketamine showed a more favorable predicted NMDA score than S-ketamine (-6.448 vs. -5.981 kcal/mol). Within the dynamically evaluated subset, NMDA end-state MM-GBSA values were -41.53 kcal/mol for R2, -28.80 kcal/mol for R11, and -42.10 kcal/mol for R14. In CES1 modeling, R14 was the only evaluated compound whose MM-GBSA became more favorable from 0 to 100 ns (-83.91 to -99.44 kcal/mol).

**Discussion:**

These results support R2 as the strongest static NMDA docking/NMDA-preference benchmark and R14 as the most internally consistent integrated computational candidate. The findings are hypothesis-generating and require synthesis and experimental validation.

## Graphical Abstract

**Figure:**
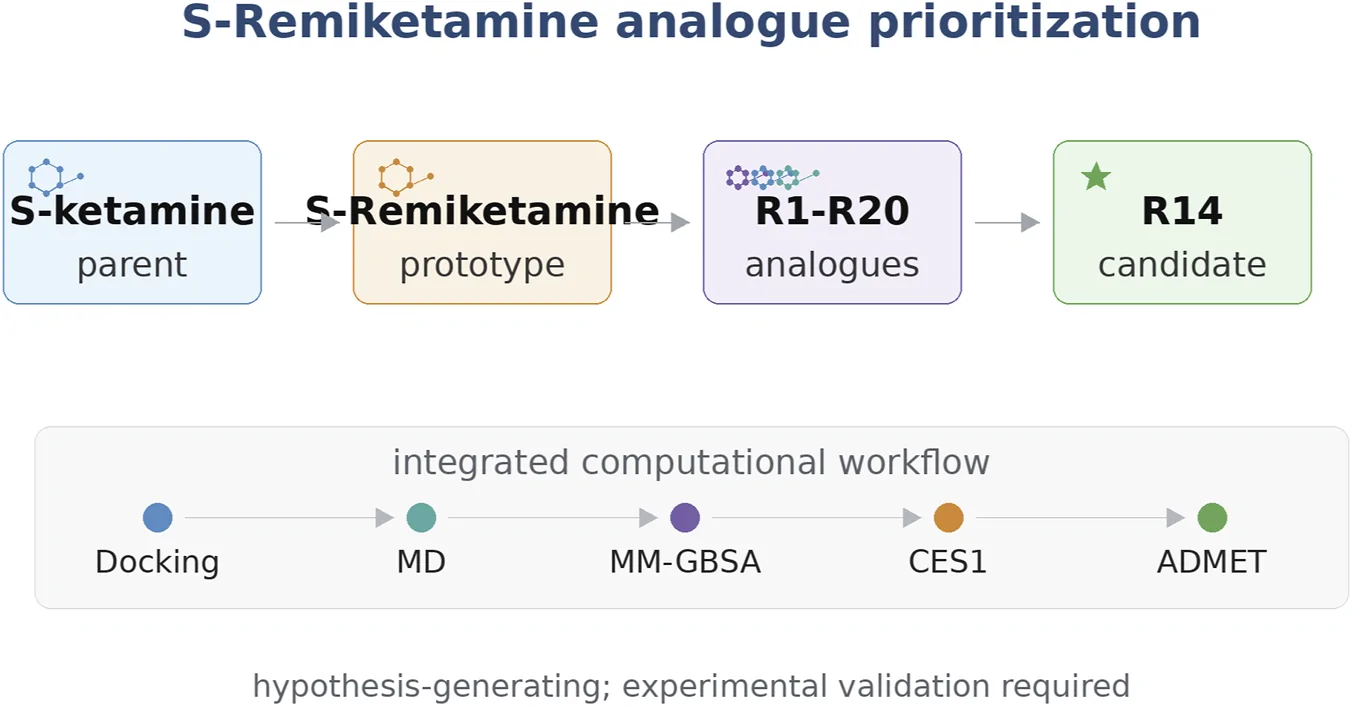

## 1 Introduction

Ketamine is an established arylcyclohexanone anesthetic and analgesic whose pharmacological profile is driven principally by non-competitive antagonism of the N-methyl-D-aspartate (NMDA) receptor, while also involving broader central nervous system target engagement (Krystal et al., 2024; Abbott et al., 2025; Villega et al., 2024). The S-enantiomer is clinically important, and recently reported human NMDA receptor structural data provide a useful framework for three-dimensional molecular modeling of ketamine-like ligands. The present work is therefore positioned within computational medicinal chemistry and computer-aided drug design, with the specific aim of using structure-based molecular modeling to prioritize analogues for future synthesis and biological validation rather than to claim experimental pharmacology.

Prior studies have explored ketamine-ester analogues and related rapidly metabolized ketamine derivatives as short-acting anesthetic concepts (Harvey et al., 2015; Jose et al., 2013; Voss et al., 2016). Those studies provide an important precedent for ester-containing ketamine-related design, but they do not remove the need to define each new stereochemical scaffold, linker architecture, and analogue library on its own structural and computational evidence. The present work therefore avoids claiming novelty for docking, molecular dynamics, or MM-GBSA methods. Instead, novelty is positioned at the level of the specific S-Remiketamine scaffold introduced here, the associated S-configured R1-R20 analogue series, and the integrated structure-based molecular modeling strategy used to compare NMDA receptor compatibility with CES1-oriented soft-drug plausibility.

From a molecular-interaction perspective, the conserved 2-chlorophenyl and cyclohexanone elements of S-ketamine provide a recognizable arylcyclohexanone framework, whereas the amine substituent is a practical perturbation point for exploring steric occupancy, polarity, hydrogen-bonding capacity, hydration, and physicochemical balance. N-substitution can also introduce ester, carbamate, acyl, oxo-linker, or dicarbonyl motifs potentially compatible with enzymatic processing. This design logic is conceptually related to soft-drug development, in which a molecule is designed to exert pharmacological action and then undergo predictable deactivation; however, such behavior can only be established experimentally, not by docking alone (Buchwald, 2020; Deng et al., 2022).

Clinically, rapidly metabolized anesthetic agents illustrate why chemically labile motifs can be attractive in anesthetic drug design. Remifentanil is distinguished among fentanyl congeners by an ester structure that supports rapid nonspecific esterase-mediated clearance, while remimazolam is an ultra-short-acting benzodiazepine hydrolyzed mainly by carboxylesterase 1 to an inactive carboxylic acid metabolite (Egan, 1995; Freyer et al., 2019). S-Remiketamine is not claimed here to share their pharmacology, clinical profile, validated metabolic rate, or safety profile; rather, remifentanil and remimazolam provide clinically familiar precedents for the broader medicinal-chemistry rationale of embedding metabolically labile motifs within anesthetic-relevant scaffolds.

The central question of this study was not whether a standard molecular-modeling workflow is itself novel, but whether systematic N-substituent variation within a conserved S-configured arylcyclohexanone core can alter the balance between static NMDA docking, trajectory-level receptor-complex support, off-target docking compatibility, and CES1 active-site structural compatibility.

Accordingly, S-Remiketamine was defined as a novel S-ketamine-derived acyloxymethyl ester prototype scaffold and expanded into a second-stage R1-R20 analogue library. The central molecular-modeling insight tested here was whether N-substituent chemistry can uncouple static NMDA docking rank from trajectory-level receptor support and CES1-pocket structural compatibility. The computational workflow integrated ligand preparation, receptor docking, cross-target docking, explicit-solvent molecular dynamics, MM-GBSA, CES1-oriented geometric assessment, ADMET prediction, and metabolite-route prediction to provide a transparent molecular-modeling assessment of scaffold-dependent behavior.

## 2 Materials and methods

### 2.1 Study design and interpretation boundaries

This study was conducted as a comparative computational medicinal-chemistry and structure-based prioritization analysis. All docking, molecular-dynamics, MM-GBSA, CES1 geometry, ADMET, and metabolite-prediction outputs were interpreted as computational and hypothesis-generating. Docking scores were used as internal target-specific ranking metrics, not as experimental binding affinities, and MM-GBSA values were used as comparative stability estimates, not as measured binding free energies or potency values.

### 2.2 Ligand set and structural curation

The ligand set comprised S-ketamine, S-Remiketamine, and analogues R1-R20, each retaining the intended S-configured arylcyclohexanone core. S-ketamine was used as the parent comparator. S-Remiketamine was defined as the novel prototype scaffold, with the N-substituent N-CH2-OCOCH3 and molecular formula C15H18ClNO3. R1-R20 represented a second-stage analogue library generated to explore controlled variation in N-substituent class, including ester, carbamate, acyl, oxo-linked, dicarbonyl-containing, and hybrid substituent types. Compound identity, structural class, molecular formula, exact mass, formal charge, and SDF-derived stereochemical descriptors are provided in Supplementary Data Sheet 2: Supplementary Table S1, with accompanying SMILES and SDF source files provided as supplementary material.

Three-dimensional ligand structures were prepared using Schrödinger LigPrep/Epik-style workflows under near-physiological protonation-state consideration, with preservation of the intended S-configuration. The deposited SDF/SMILES files are compound-identity records and show formal charge 0 for all entries; they should not be interpreted as a full protonation-state ensemble. Ligand preparation incorporated Epik-style enumeration/selection under near-physiological conditions, and all docking and MD interpretations are therefore conditional on the prepared ligand states used in the workflow (Schrodinger, LLC, 2022). For Reviewer 2-oriented structural visualization, local-minimum 3D conformers were additionally regenerated from the deposited SDF/SMILES identity files using RDKit ETKDGv3 embedding followed by MMFF/UFF local minimization. These reviewer-response conformers are provided in Supplementary Data Sheet 7 and are not experimental structures.

### 2.3 Protein structures and receptor preparation

The primary pharmacological model was the human NMDA receptor structure PDB 7EU8, selected because it is a ketamine-bound human NMDA receptor structural template directly aligned with the parent pharmacological scaffold (Zhang et al., 2021). Use of a single NMDA structural template was deliberate for internal comparability across the ligand series, but it is also a limitation because alternative NMDA conformations, subunit states, membrane contexts, and channel states were not exhaustively sampled. Off-target proteins represented systems relevant to serotonergic, dopaminergic, opioid, psychotomimetic, and cardiotoxicity-associated liabilities: SERT (PDB 5I6X), DAT (PDB 9EO4), mu-opioid receptor (PDB 5C1M), kappa-opioid receptor (PDB 9D61), sigma-1 receptor (PDB 6DJZ), and the hERG potassium channel (PDB 5VA1) (Coleman et al., 2016; Huang et al., 2015; Nielsen et al., 2024; Schmidt et al., 2018; Tyson et al., 2025; Wang and MacKinnon, 2017). Human carboxylesterase 1 (CES1; PDB 1MX9) was used as an esterase-compatibility model (Bencharit et al., 2003; Her and Zhu, 2020). PDB identifiers were used as structural templates for comparative modeling and docking only; the selected structures differ in experimental method, resolution, ligand state, species where applicable, and conformational context. Protein structures were prepared using Schrödinger Protein Preparation Wizard-style procedures and established protein/ligand-preparation principles (Sastry et al., 2013), including bond-order assignment, hydrogen addition, protonation-state optimization, hydrogen-bond network optimization, restrained minimization, and grid generation around co-crystallized ligands or defined active-site regions.

### 2.4 Docking workflow and derived NMDA-preference metrics

The full ligand set was docked to NMDA and the off-target panel using Glide-style receptor-grid docking, with standard-precision screening followed by extra-precision refinement of top-ranking poses as described in the source records and Supplementary Methods (Friesner et al., 2004; Friesner et al., 2006). Binding-site definitions were based on the co-crystallized ligand/active-site region of each prepared structural template or, for CES1, the catalytic Ser221-centered active-site environment. CES1 source records identify the grid as centered on the Ser221 catalytic region, with reported centre coordinates approximately −0.711080, −31.422480, −16.809360 and a 20 × 20 × 20 A box. The available source set supports use of prepared/minimized receptor grids with flexible ligand sampling; it does not support claiming induced-fit docking or a defined flexible amino-acid side-chain set, and no such residue-flexible docking claim is made. For NMDA, exact numerical grid-center coordinates and full run-level search parameters were not present in the public shareable dataset; native job records are retained by the authors and may be provided for editorial inspection where permitted. Reference ligands in the source records were used for orientation and plausibility checks, not for experimental validation.

Mean predicted off-target compatibility was summarized using mean Delta (off-target - NMDA), calculated as the average of off-target docking score minus NMDA docking score across SERT, DAT, mu-opioid receptor, kappa-opioid receptor, sigma-1 receptor, and hERG. A larger positive value indicates weaker predicted mean off-target binding relative to NMDA within this internal comparative framework. The full docking matrix is provided in Supplementary Data Sheet 2: Supplementary Table S2.

### 2.5 Selection of compounds for dynamic analysis

R2, R11, and R14 were selected for advanced molecular-dynamics and MM-GBSA analysis because they represented distinct design roles. R2 was the strongest static NMDA docking/NMDA-preference benchmark. R11 was a polar glycolyl-linker analogue used as a mechanistic comparator. R14 was a dicarbonyl-containing acetoacetyl analogue selected for integrated dynamic and CES1-oriented analysis because it combined competitive NMDA docking with low predicted off-target compatibility and a substituent motif suitable for esterase-pocket evaluation. This tiered workflow was deliberate: the full library was first screened by docking and off-target profiling, after which R2, R11, and R14 were selected for deeper trajectory-level and MM-GBSA assessment as representative compounds occupying distinct roles in the design landscape.

### 2.6 Molecular dynamics, MM-GBSA, and CES1 geometric assessment

NMDA receptor-ligand complexes for R2, R11, and R14 were simulated in explicit solvent using Desmond-style molecular dynamics under matched comparative conditions (Bowers et al., 2006). Source-methodology records describe orthorhombic periodic solvent boxes with at least a 10 A buffer around the protein, charge neutralization and physiological salt supplementation with 0.15 M NaCl, NPT production dynamics, a 2 fs integration timestep, 9.0 A short-range non-bonded cutoffs, Particle Mesh Ewald treatment of long-range electrostatics, and constraints on bonds involving hydrogen atoms. The primary comparative NMDA simulations were 100 ns receptor-ligand complex simulations performed without an explicit membrane bilayer; the prepared 7EU8 receptor-complex model was therefore not treated as a complete physiological membrane-embedded NMDA channel model. A source audit of the uploaded MD simulation reports, trajectory-analysis reports, PL-contact files, and time-point trajectory images identified ligand-bound NMDA and CES1 complex simulations for R2, R11 and R14, but no matched apo/free-receptor production MD dataset. Accordingly, matched free-receptor MD was not analysed in this study, and ligand-induced receptor conformational changes relative to apo receptor trajectories are not claimed. Source-report RMSD/RMSF panels for NMDA-R2, NMDA-R11 and NMDA-R14 are now provided in Supplementary Data Sheet 7: Supplementary Figure S7. NMDA MM-GBSA source methods describe analysis of stabilized final-trajectory snapshots from the last 20 ns at 100 ps sampling, corresponding to approximately 200 frames; MM-GBSA values are interpreted only as comparative energy estimates within this workflow (Li et al., 2011; Du et al., 2011).

Reviewer-requested electrostatic and binding-site complementarity visualizations were generated from the source-supported structure and contact data. Ligand electrostatic-potential-like surface maps were generated from the deposited SDF structures using RDKit 3D coordinates and Gasteiger partial charges. Binding-site complementarity was summarized from uploaded Desmond PL-Contacts files by calculating residue-level contact occupancy across available trajectory frames for hydrogen-bond/water-bridge/aromatic and hydrophobic interaction classes. These analyses are reported in Supplementary Data Sheet 7: Supplementary Figure S9 and are interpreted as qualitative structural visualization only.

CES1 complexes of R2, R11, and R14 were also subjected to matched explicit-solvent molecular dynamics. Source reports describe 100 ns Desmond simulations with OPLS4, TIP3P water, charge neutralization, 0.15 M NaCl, equilibration, and trajectory storage at 100 ps intervals, yielding 1,000 stored frames. CES1 analyses evaluated ligand stability in the esterase pocket, persistence of structurally compatible orientations near the Ser221-centered catalytic environment, and geometric descriptors relevant to serine-hydrolase-like nucleophilic approach. Key observables included Ser221 O-gamma to ligand-carbonyl carbon distance, attack-angle metrics, catalytic-triad hydrogen-bond occupancy, oxyanion-hole hydrogen-bond occupancy, and water-bridge occupancy. CES1 docking source records describe a grid centered on the catalytic serine region and post-docking geometric screening using Ser221, Gly142/Gly143, Ala222, His468, and Glu354 interactions. Source-report RMSD/RMSF panels for CES1-R2, CES1-R11 and CES1-R14 are now provided in Supplementary Data Sheet 7: Supplementary Figure S8. CES1 MM-GBSA source methods describe representative-frame and end-state reporting, including representative frames every 10 ns and 0/100 ns summaries; these values are interpreted as comparative energy estimates rather than measured binding free energies, catalytic rates, or hydrolysis evidence.

### 2.7 ADMET, metabolite prediction, and data availability

Selected compounds were assessed using *in silico* ADMET and metabolite-prediction workflows. SwissADME-style descriptors included molecular weight, topological polar surface area, lipophilicity, hydrogen-bond donor/acceptor counts, qualitative gastrointestinal absorption, blood-brain-barrier/BOILED-Egg interpretation, P-glycoprotein prediction, and CYP-related prediction flags (Daina et al., 2017). BioTransformer-style outputs were used to summarize explicitly recoverable predicted metabolite routes. These predictions are reported as molecular-modeling context only and not as pharmacokinetic or toxicological validation (Djoumbou-Feunang et al., 2019).

### 2.8 Reproducibility and data traceability

Compound identity was cross-checked using Supplementary Data Sheet 2: Supplementary Table S1, the isomeric SMILES file, and the SDF structure archive. For the key advanced compounds, SDF-derived formulae and exact masses were independently checked from the supplied structure files: R2, C17H22ClNO3, exact MW 323.128821 Da; R11, C15H18ClNO3, exact MW 295.097521 Da; and R14, C17H20ClNO3, exact MW 321.113171 Da. The complete NMDA/off-target docking matrix is provided in Supplementary Data Sheet 2: Supplementary Table S2, dynamic NMDA and CES1 summaries are provided in Supplementary Data Sheet 2: Supplementary Tables S3-S4, and ADMET/metabolite-prediction summaries are provided in Supplementary Data Sheet 2: Supplementary Table S5. Figure-level numerical source data are provided in Supplementary Data Sheet 3. Machine-readable SMILES and SDF files are provided in Supplementary Data Sheets 4 and 5. The minimal supporting dataset has also been deposited in Zenodo and is available at https://doi.org/10.5281/zenodo.21725205.

## 3 Results

### 3.1 S-Remiketamine scaffold definition and R-series design logic

**FIGURE 1:**
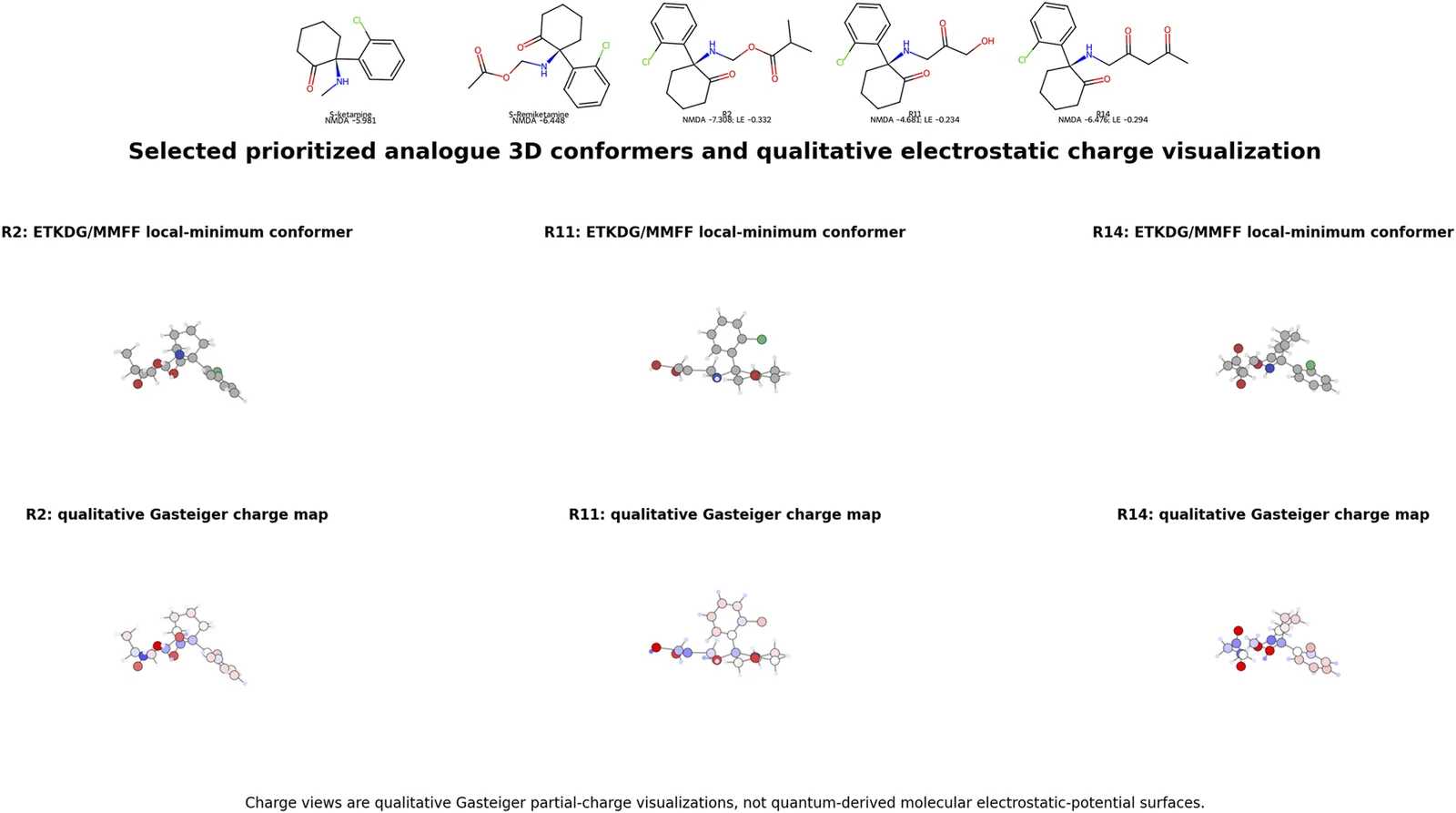
Two-stage design logic, representative structures, selected local-minimum 3D conformers, and qualitative electrostatic charge visualization. The upper panel shows representative 2D structures for S-ketamine, S-Remiketamine, R2, R11, and R14, including NMDA docking score and docking ligand efficiency where applicable. The lower panels show RDKit ETKDG/MMFF local-minimum conformers for R2, R11, and R14 and qualitative Gasteiger partial-charge maps. Additional reviewer-requested electrostatic and binding-site complementarity analysis is provided in Supplementary Data Sheet 7: Supplementary Figure S9. The ligand maps are molecular electrostatic-potential-like surface visualizations generated from deposited SDF structures using RDKit 3D coordinates and Gasteiger partial charges, and the binding-site complementarity panels summarize source-derived Desmond PL-contact occupancies for polar/aromatic and hydrophobic residues.

The design pathway was organized around a conserved S-configured arylcyclohexanone core. S-Remiketamine was introduced as an acyloxymethyl ester prototype scaffold derived from S-ketamine by rational N-substitution. The R1-R20 library then explored systematic substituent changes across carbamate, ester, acyl, oxo-linked, dicarbonyl-containing, acid-containing, and hybrid motifs. This design makes the series interpretable as a structured perturbation library rather than a collection of unrelated compounds. Relative to the parent scaffold, the R-series deliberately increased the range of molecular weight, topological polar surface area, hydrogen-bond acceptor/donor capacity, lipophilicity, steric bulk, and hydrolytically labile or carbonyl-rich substituent chemistry. The complete descriptor and docking ligand-efficiency matrix is provided in Supplementary Data Sheet 7: Supplementary Table S10, and the full local-minimum 3D conformer panel for S-ketamine, S-Remiketamine, and R1-R20 is provided as Supplementary Figure S1. The two-stage design logic, representative structures, selected local-minimum 3D conformers, and qualitative charge maps are shown in Figure 1.

The R-series can therefore be read structurally as follows: R1/R5/R20 test carbamate or carbamate-like donor/acceptor motifs; R2/R9 increase hydrophobic or aryl/acyl bulk; R3/R4/R16/R18 probe smaller or halogenated ester variants; R7 represents a hydroxymethylamine-type polar analogue; R8/R12/R14 enrich carbonyl density and potential downstream polar-metabolite routes; R11/R13/R15 introduce more polar oxo, acid, or amide-containing linkers; and R19 probes a simpler oxo-alkyl chain. These substitutions do not prove improved biology, but they explain how N-substituent chemistry was used to vary molecular properties and prioritize R2, R11, and R14 for deeper comparative analysis.

The public structural files support this scaffold definition. The SMILES/SDF source data identify S-Remiketamine as C15H18ClNO3 with formal charge 0, S-configured stereochemistry, and the isomeric SMILES CC(=O)OCN[C@]1(c2ccccc2Cl)CCCCC1=O. The same molecular formula is shared by R11, but the source files distinguish the two structures by connectivity, confirming that formula identity alone is insufficient for structural identity. For R14, the final SDF-derived formula and exact mass used in this submission are C17H20ClNO3 and 321.113171 Da.

### 3.2 NMDA docking ranks R2 highest, while S-Remiketamine shows a more favorable predicted score than the parent comparator

**FIGURE 2:**
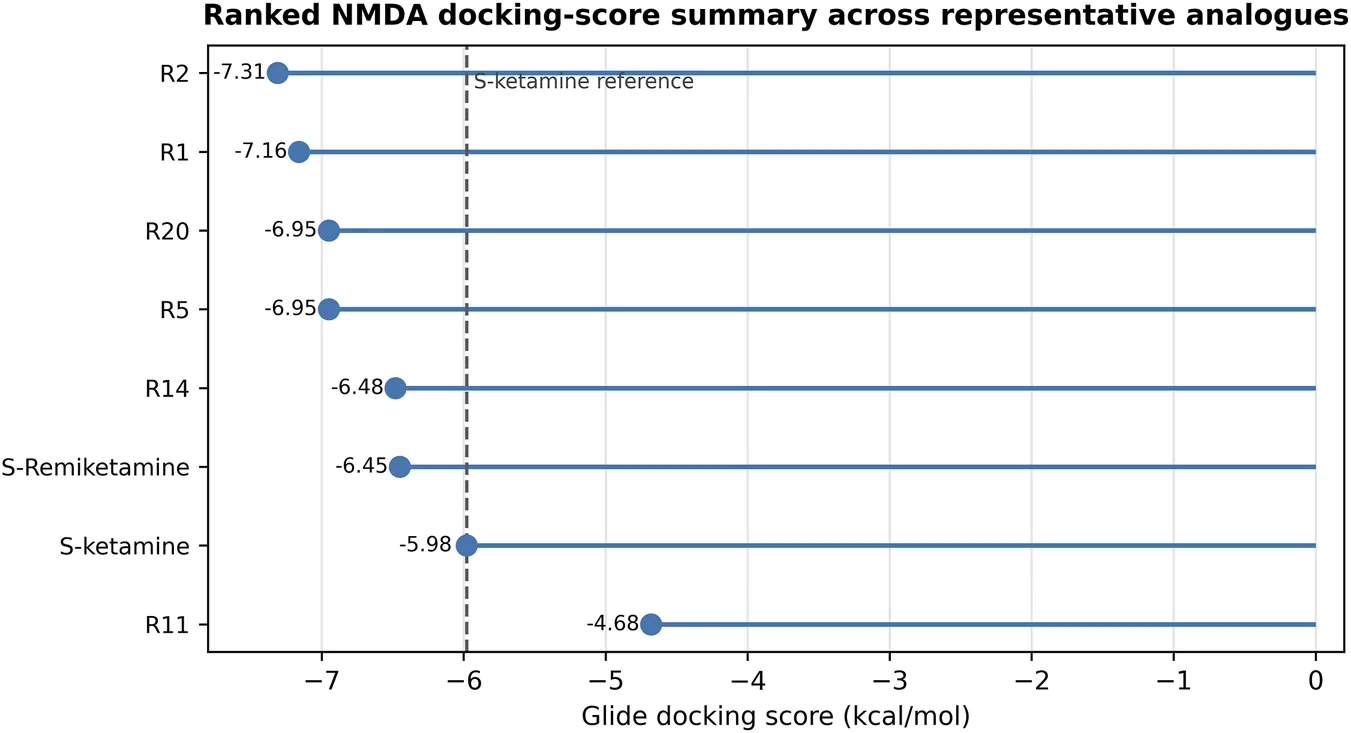
Ranked NMDA docking-score summary. More negative values indicate stronger predicted binding within the same receptor model. The dashed reference line indicates the S-ketamine docking score. Values are computational summary metrics from the source-data file and are not experimental biological replicates.

Static NMDA docking gave Glide scores of −5.981 kcal/mol for S-ketamine and −6.448 kcal/mol for S-Remiketamine, indicating a more favorable predicted NMDA docking score for the prototype relative to the parent comparator within this internal model. Among representative analogues, the strongest NMDA docking scores were observed for R2 (−7.308 kcal/mol), R1 (−7.162 kcal/mol), R5 and R20 (−6.949 kcal/mol), R14 (−6.476 kcal/mol), S-Remiketamine (−6.448 kcal/mol), S-ketamine (−5.981 kcal/mol), and R11 (−4.681 kcal/mol). The ranked NMDA docking-score summary is shown in Figure 2.

To address ligand-size dependence, docking ligand efficiency was calculated as NMDA docking score divided by heavy-atom count. This metric supported R2 as the strongest selected static docking benchmark among the advanced subset (R2 −0.332 kcal/mol/heavy atom), while R14 remained competitive (−0.294 kcal/mol/heavy atom) and R11 remained weaker (−0.234 kcal/mol/heavy atom). The complete ligand-efficiency table for S-ketamine, S-Remiketamine, and R1-R20 is provided in Supplementary Data Sheet 7: Supplementary Table S10. These ligand-efficiency values remain docking-derived internal ranking metrics and are not experimental efficiencies or affinities.

These values support R2 as the strongest static NMDA docking/NMDA-preference benchmark. However, the result also confirms a major limitation: several compounds have modest predicted docking scores and should not be described as strong NMDA binders. Accordingly, weak-scoring compounds were interpreted as low-priority or mechanistic comparators rather than as promising pharmacological candidates.

### 3.3 Cross-target docking supports internal NMDA-preference signals but not experimental selectivity

**FIGURE 3:**
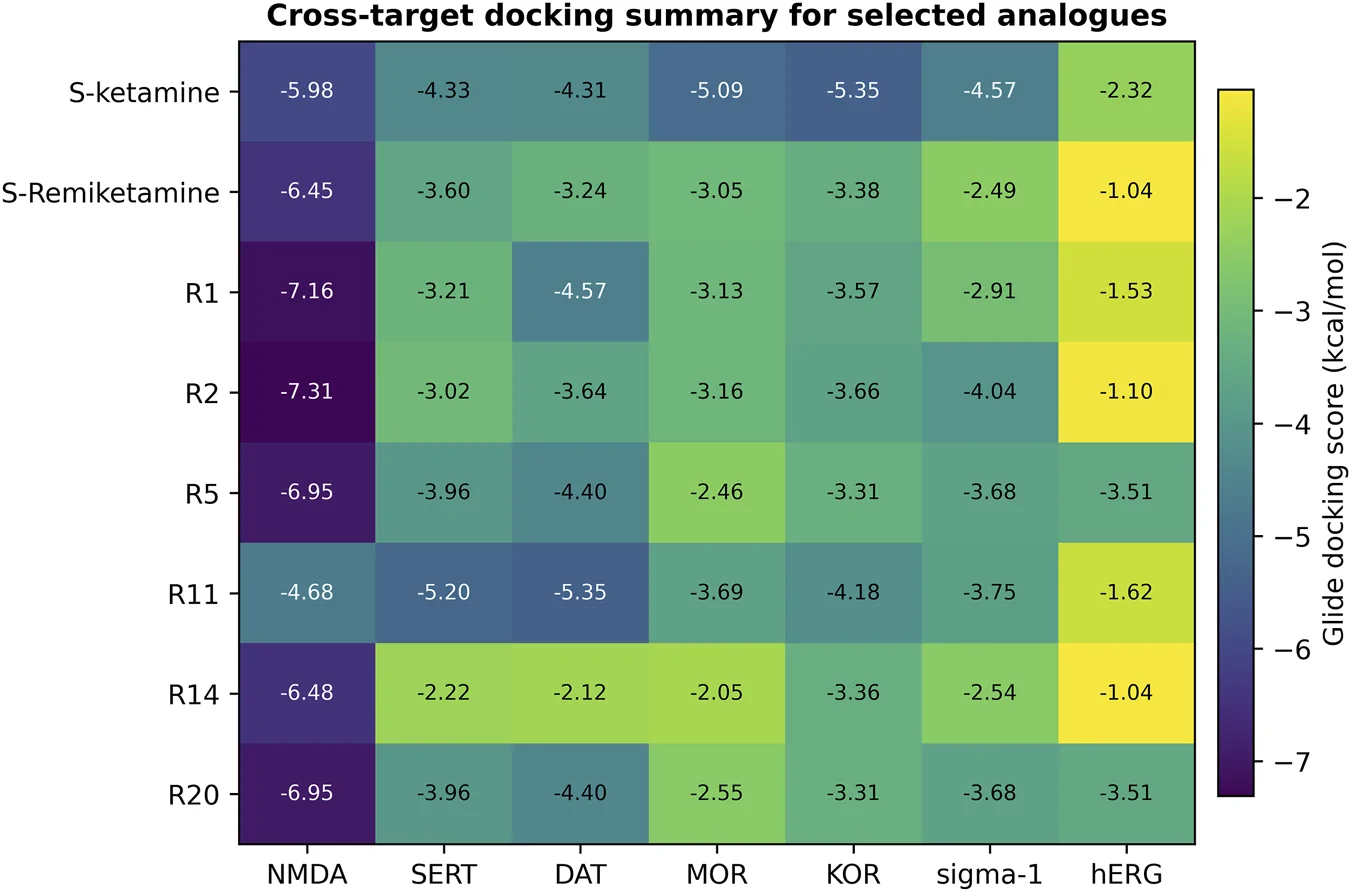
Cross-target docking summary. The heat map reports docking scores across NMDA receptor, SERT, DAT, mu-opioid receptor, kappa-opioid receptor, sigma-1 receptor, and hERG. Values should not be compared as experimental affinities across unrelated proteins.

The cross-target panel suggested substituent-dependent differences in predicted off-target compatibility. For representative analogues, mean Delta (off-target - NMDA) was 1.652 kcal/mol for S-ketamine, 3.648 kcal/mol for S-Remiketamine, 4.008 kcal/mol for R1, 4.203 kcal/mol for R2, 3.398 kcal/mol for R5, 0.716 kcal/mol for R11, 4.256 kcal/mol for R14, and 3.383 kcal/mol for R20. R14 therefore showed the largest internal NMDA-preference metric among the representative set, whereas R2 remained the strongest static NMDA docking hit. The cross-target docking heat map is shown in Figure 3.

These cross-target results are computational NMDA-preference/off-target-compatibility signals only. They should not be interpreted as evidence of transporter inactivity, opioid inactivity, sigma-1 inactivity, hERG safety, or clinical safety. The off-target models provide a triage framework for prioritization, not a substitute for receptor, transporter, or electrophysiology assays.

### 3.4 NMDA molecular dynamics separates static docking rank from dynamic support

**FIGURE 4:**
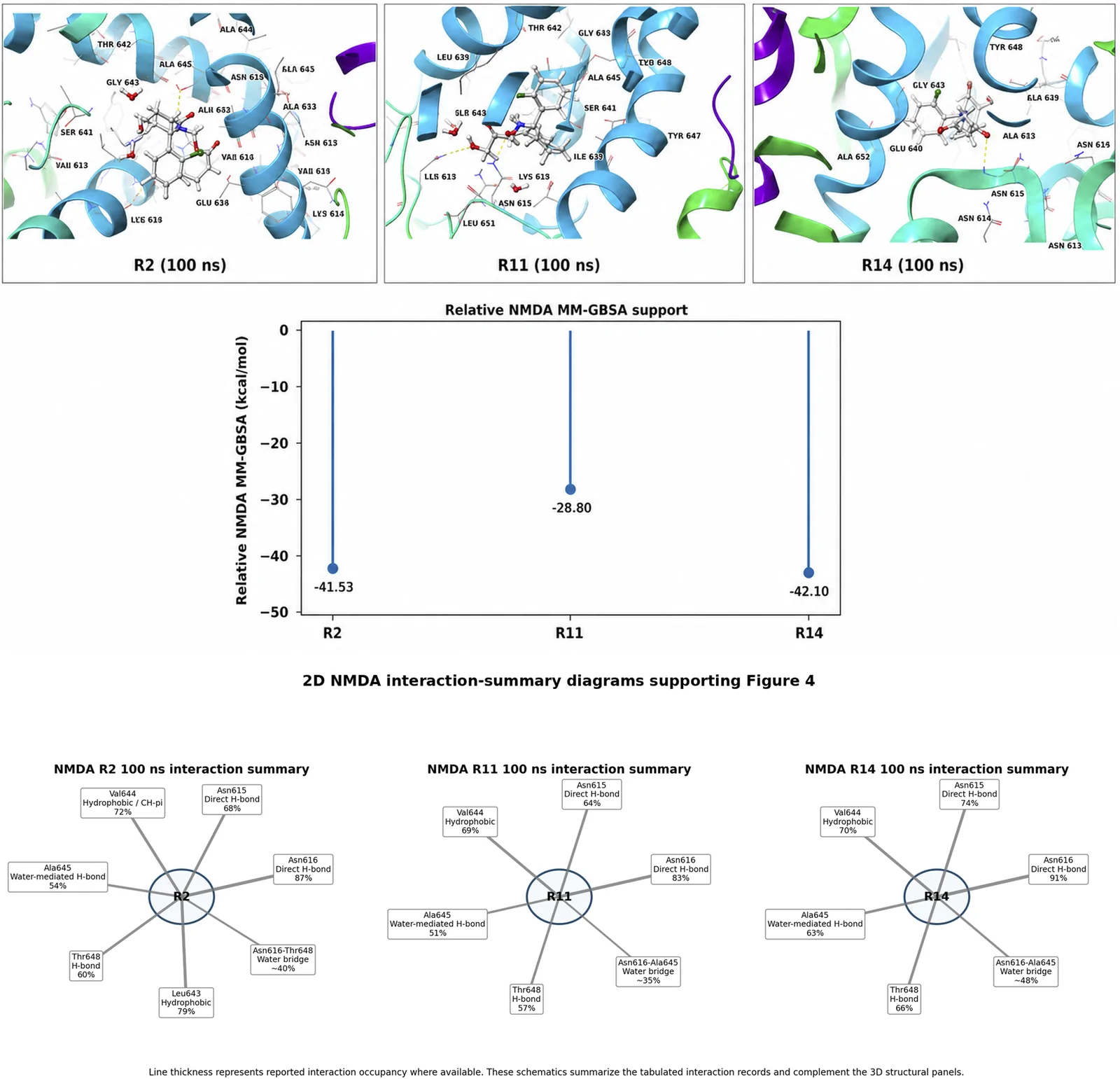
Comparative NMDA receptor molecular-dynamics analysis with 2D interaction-summary diagrams. Representative 100 ns snapshots are shown for R2, R11, and R14 with comparative end-state NMDA MM-GBSA values. The added schematic panel summarizes the tabulated 100 ns NMDA interaction occupancies for each compound to improve readability of intermolecular contacts. These outputs are comparative computational metrics and interaction summaries, not experimental potency measurements.

R2, R11, and R14 were evaluated by explicit-solvent NMDA molecular dynamics as representatives of the strongest static docking/NMDA-preference benchmark, a polar-linker comparator, and a dicarbonyl-containing advanced analogue, respectively. Comparative NMDA end-state MM-GBSA values were −41.53 kcal/mol for R2, −28.80 kcal/mol for R11, and −42.10 kcal/mol for R14. Within this limited dynamic subset, R14 retained the most favorable end-state MM-GBSA support, whereas R2 remained the strongest static docking benchmark and R11 remained the weakest NMDA dynamic comparator. Representative NMDA molecular-dynamics snapshots and comparative end-state MM-GBSA values are shown in Figure 4.

Residue-contact summaries supported persistent polar and water-mediated interactions in the Asn615/Asn616 region, with hydrophobic support from residues such as Leu643, Val644, and Ala645. R14 showed the highest reported Asn616 direct hydrogen-bond occupancy (91%) and higher water-mediated Ala645 interaction frequency than R2 or R11 in the submitted interaction summaries. These findings provide comparative dynamic support, not experimental receptor potency or channel-gating evidence.

### 3.5 CES1 modeling prioritizes R14 for esterase-pocket structural compatibility

**FIGURE 5:**
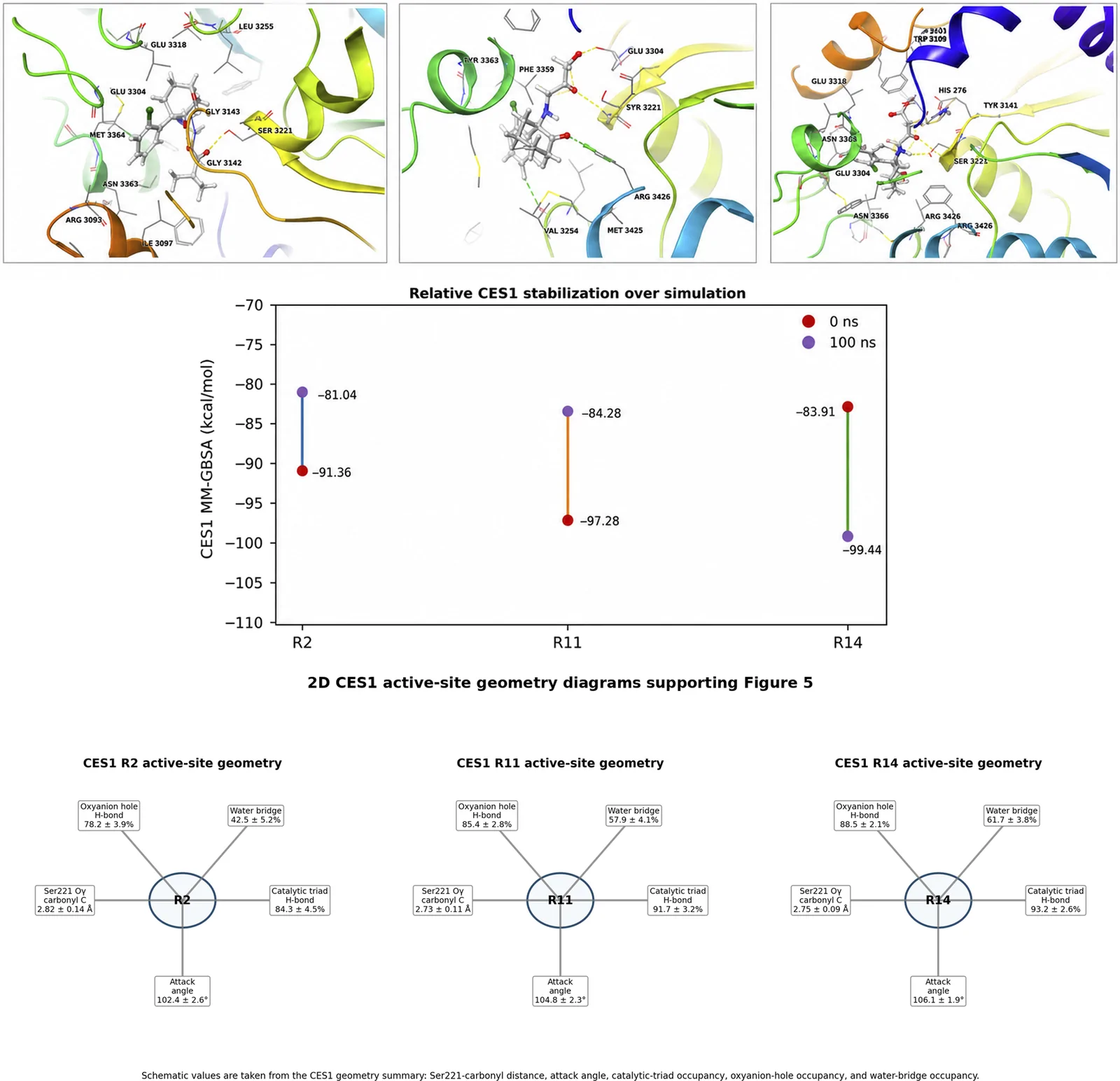
Comparative CES1 compatibility analysis with 2D active-site geometry diagrams. Upper panels show representative 100 ns CES1 snapshots for R2, R11, and R14; comparative CES1 MM-GBSA values are shown at 0 and 100 ns. The added schematic panel summarizes Ser221-carbonyl distance, attack angle, catalytic-triad hydrogen-bond occupancy, oxyanion-hole occupancy, and water-bridge occupancy. These analyses support comparative assessment of CES1-compatible ligand orientations and should not be interpreted as hydrolysis-rate or turnover measurements.

CES1 docking, molecular dynamics, MM-GBSA, and geometric analyses were used to compare whether R2, R11, and R14 could adopt structurally compatible orientations near the Ser221-centered esterase environment. CES1 MM-GBSA changed from −91.36 to −81.04 kcal/mol for R2, from −97.28 to −84.28 kcal/mol for R11, and from −83.91 to −99.44 kcal/mol for R14 between 0 and 100 ns. R14 was therefore the only compound whose CES1 MM-GBSA became more favorable over the simulation interval. Representative CES1 snapshots and comparative MM-GBSA evolution are shown in Figure 5.

The structured CES1 geometry workbook reported Ser221 O-gamma to carbonyl-carbon distances of 2.82 ± 0.14 Å for R2, 2.73 ± 0.11 Å for R11, and 2.75 ± 0.09 Å for R14, with attack-angle metrics of 102.4 ± 2.6 degrees, 104.8 ± 2.3 degrees, and 106.1 ± 1.9 degrees, respectively. R14 also had the highest catalytic-triad hydrogen-bond occupancy (93.2% ± 2.6%), oxyanion-hole hydrogen-bond occupancy (88.5% ± 2.1%), and water-bridge occupancy (61.7% ± 3.8%). These results support structural compatibility with CES1 engagement but do not establish hydrolysis rate, enzymatic turnover, or plasma clearance.

### 3.6 Predictive ADMET and metabolite-route analyses provide context only

R2, R11, and R14 remained within standard small-molecule descriptor ranges in the final source set. R2 and R14 were predicted to have CNS-oriented descriptor profiles, whereas R11 behaved as a more polar comparator with reduced predicted blood-brain-barrier penetration in the source output. R14 had exact MW 321.1 Da, predicted pKa 7.6, TPSA 59.4 Å^2^, LogP 2.4, one hydrogen-bond donor, and four hydrogen-bond acceptors. Predicted metabolite-route enumeration was most explicit for R14 and included glucuronidation, hydroxylation, carbonyl reduction, and hydrolytic cleavage routes.

These ADMET and metabolite predictions are supportive context only. They do not validate blood-brain-barrier penetration, metabolic rate, pathway dominance, metabolite formation, metabolite inactivity, excretion route, toxicology, pharmacokinetics, or safety.

### 3.7 Integrated prioritization

**FIGURE 6:**
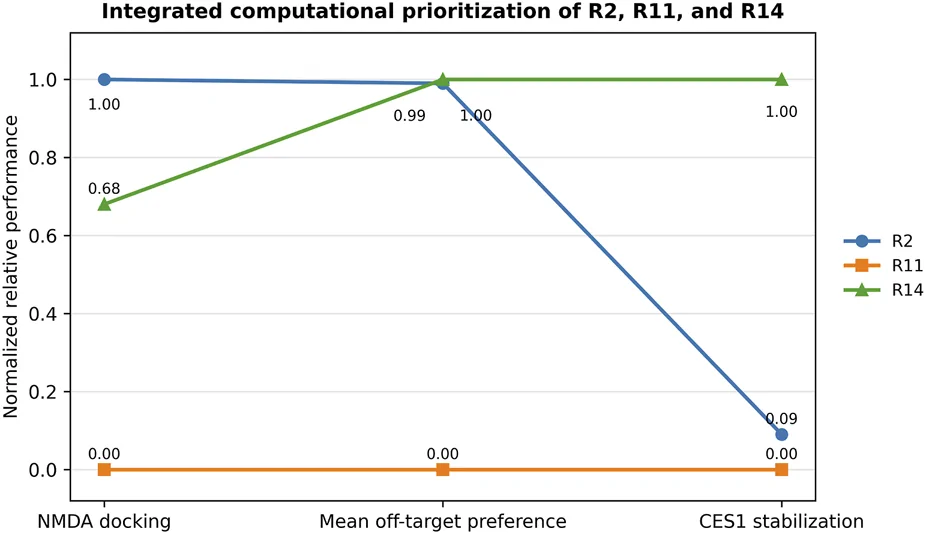
Integrated structure-based prioritization. Normalized comparative performance is shown for R2, R11, and R14 across NMDA docking, internal mean off-target preference metric, and CES1 stabilization. R2 remains the strongest static docking/NMDA-preference benchmark, whereas R14 shows the most internally consistent integrated computational profile overall.

The principal molecular-modeling insight of the integrated workflow is that static docking rank and integrated candidate priority can diverge. R2 was the strongest static NMDA docking/NMDA-preference benchmark. R11 was a mechanistic polar-linker comparator. R14 was prioritized as the most internally consistent advanced analogue when competitive NMDA docking, favorable NMDA dynamic MM-GBSA support, reduced predicted off-target compatibility, CES1-pocket stabilization, and predicted ADMET/metabolite context were considered together. The integrated prioritization profile is shown in Figure 6, and the compound-level interpretation is summarized in Table 1.

**TABLE 1:** Integrated interpretation of R2, R11, and R14.

Compound	Most defensible role	Key supporting observations	Interpretation boundary
R2	Top static docking/NMDA-preference benchmark	Best NMDA docking score (−7.308 kcal/mol); favorable NMDA MM-GBSA (−41.53 kcal/mol); strong mean Delta (off-target - NMDA)	Not the integrated CES1/dynamic candidate; docking is not experimental potency
R11	Mechanistic polar-linker comparator	Weak NMDA docking (−4.681 kcal/mol) and weakest NMDA MM-GBSA (−28.80 kcal/mol); useful polar comparator for the advanced subset.	Should not be presented as a prioritized candidate
R14	Prioritized integrated computational candidate	Competitive NMDA docking (−6.476 kcal/mol); NMDA MM-GBSA broadly comparable to R2 and more favorable than R11 (−42.10 kcal/mol); largest representative mean Delta (off-target - NMDA); CES1 MM-GBSA improved from −83.91 to −99.44 kcal/mol	Hypothesis-generating prioritization only; requires synthesis and experimental validation before biological conclusions

## 4 Discussion

### 4.1 Principal interpretation

This manuscript should be read as a computational medicinal-chemistry and structure-based molecular-modeling study rather than as a claim of validated pharmacology. The data introduce the specific S-Remiketamine scaffold and derived analogue library, show how substituent chemistry changes predicted NMDA and CES1 structural behavior, and identify which analogues merit future synthesis and experimental testing.

The strongest single-target docking result was R2. However, when dynamic NMDA support, cross-target docking, CES1 structural compatibility, MM-GBSA evolution, and predictive ADMET/metabolite data were considered together, R14 emerged as the most defensible integrated computationally prioritized candidate. This divergence is scientifically useful because it shows that in this S-configured arylcyclohexanone series, N-substituent chemistry can uncouple static receptor docking rank from broader dynamic and esterase-pocket behavior.

### 4.2 Novelty and molecular-modeling insight

The novelty of this study should not be framed as novelty of docking, molecular dynamics, MM-GBSA, or ADMET prediction. Those are established methods. The novelty lies in introducing and structurally defining the specific S-Remiketamine scaffold, expanding it into a stereochemically consistent R1-R20 analogue series, and comparing how N-substituent chemistry modifies NMDA receptor docking, explicit-solvent dynamic support, off-target docking compatibility, and CES1-oriented structural behavior.

To the authors’ knowledge, this is the first report to define the specific S-Remiketamine chemical scaffold and the specific S-Remiketamine-derived analogue series described by the submitted SDF, SMILES, and compound-identity records. Prior literature includes related ketamine-ester analogues and rapidly metabolized ketamine-related concepts; therefore, the broader ester/prodrug or soft-drug concept is not claimed as new.

The intended analogy to clinically established rapidly metabolized anesthetic agents should therefore be interpreted cautiously. Remifentanil and remimazolam demonstrate that ester-enabled design can be clinically useful when supported by pharmacology, metabolism, and safety data (Egan, 1995; Freyer et al., 2019). The present study does not establish that S-Remiketamine will behave like either agent. Instead, it defines a chemically distinct S-ketamine-derived scaffold and prioritizes analogues for the experimental work that would be required to test whether an ester-containing arylcyclohexanone can achieve a favorable short-acting profile.

### 4.3 Interpretation of weak and modest docking scores

Several compounds in the series showed weak or modest docking scores. This is not a problem if interpreted honestly. Low-scoring compounds help define the design landscape and identify structures that should not be prioritized. The manuscript therefore avoids describing the entire analogue series as promising. Instead, it separates low-priority structures, mechanistic comparators, static docking benchmarks, and integrated candidates.

Even for the better-scoring analogues, docking scores should not be converted into claims of potency. Docking score differences can be sensitive to receptor preparation, binding-site definition, protonation state, scoring function, and pose selection. The most appropriate interpretation is relative ranking within a defined computational workflow. Experimental receptor binding and functional assays remain essential.

### 4.4 Why R14 is the integrated candidate rather than simply the best docking hit

R14 was not the strongest static NMDA docking hit. Its prioritization depends on the integrated nature of the workflow: competitive NMDA docking, NMDA end-state MM-GBSA support broadly comparable to R2 and more favorable than R11, the highest representative mean Delta (off-target - NMDA), improvement in CES1 MM-GBSA over 0–100 ns, and favorable CES1 geometric descriptors. This integrated profile makes R14 a rational computationally prioritized candidate for future synthesis and experimental validation, but not an experimentally validated pharmacological lead.

### 4.5 Limitations

The study has several important limitations. First, no experimental NMDA receptor binding, electrophysiological, functional, enzymatic, plasma-stability, metabolite-identification, toxicology, or pharmacokinetic data are provided. Second, docking and MM-GBSA are approximate computational methods and should not be converted into potency or efficacy claims. Third, the NMDA analysis used a single ketamine-relevant structural template and did not exhaustively sample alternative NMDA conformations, receptor states, membrane-embedded gating states, or subunit contexts. Fourth, matched free-receptor MD simulations were not included in the curated dataset; therefore, ligand-induced receptor conformational effects relative to apo receptor trajectories cannot be inferred. Fifth, source-report RMSD/RMSF plots are now provided for the ligand-bound NMDA and CES1 systems in Supplementary Data Sheet 7, but the underlying numeric time-series were not supplied as independent CSV files for complete re-plotting. Sixth, in response to the reviewer request for electrostatic potential and binding-site complementarity visualization, Supplementary Figure S9 adds ligand electrostatic-potential-like surface maps derived from deposited SDF structures using Gasteiger partial charges and source-derived binding-site contact-complementarity heatmaps from Desmond PL-Contacts files. These are qualitative visualization and contact-occupancy analyses rather than quantum-mechanical MEP calculations or new quantitative binding-free-energy calculations. These boundaries are essential to prevent overinterpretation of the computational outputs.

### 4.6 Future validation

The next experimental steps should include synthesis of S-Remiketamine and prioritized R-series compounds, stereochemical and purity verification, independent structural confirmation, NMDA receptor binding and functional testing, off-target pharmacology, hERG electrophysiology, CES1 hydrolysis assays, plasma and microsomal stability studies, metabolite identification, and early pharmacokinetic/pharmacodynamic evaluation. Until those studies are performed, the present dataset should be used for computational prioritization and hypothesis generation only.

## 5 Conclusion

This study introduces the specific S-Remiketamine scaffold as a novel S-ketamine-derived acyloxymethyl ester prototype and uses it to anchor a second-stage R1-R20 library of S-configured arylcyclohexanone analogues. Static NMDA docking identified R2 as the strongest docking/NMDA-preference benchmark, whereas integrated structure-based molecular modeling prioritized R14 when NMDA dynamic support, off-target docking, CES1 structural compatibility, MM-GBSA evolution, and ADMET/metabolite context were considered together. The work provides a transparent structure-based basis for future synthesis and experimental validation, but does not establish receptor potency, hydrolysis rate, enzymatic turnover, plasma clearance, pharmacokinetics, efficacy, or safety. The most appropriate interpretation is therefore a computational medicinal-chemistry prioritization framework, not biological proof of a drug candidate.

## Data Availability

The datasets presented in this study can be found in Zenodo and in the article/Supplementary Material. The Zenodo repository is available at: https://doi.org/10.5281/zenodo.21725205. The deposited dataset includes curated figure source data, isomeric SMILES identifiers, SDF structure files for S-ketamine, S-Remiketamine and R1-R20 analogues, supplementary tables, supplementary methods, revised figures, graphical abstract, and a README/data dictionary. Supplementary Data Sheet 7 is provided with the article Supplementary Material and contains additional reviewer-response structural and trajectory-analysis addenda. Public protein structures analyzed in the study were obtained from the Protein Data Bank using the PDB identifiers reported in the manuscript. Native computational job records and additional source reports may be provided for editorial inspection where permitted by the submission system. No new custom software or algorithm is reported in this study.
