# Supplementary material for: Structure-based molecular modeling of a novel S-Remiketamine scaffold and derived arylcyclohexanone analogues: NMDA receptor dynamics, MM-GBSA ranking, and CES1-Oriented structural compatibility
Source: Front Chem. 2026 Sep 3;14:1922077. doi: 10.3389/fchem.2026.1922077 (PMC13582596; doi:10.3389/fchem.2026.1922077)
Supplement: Supplementary file 1 [file DataSheet5.pdf]

Supplementary Data Sheet 6

README and Data Dictionary for Frontiers in Chemistry Supplementary Material

Frontiers in Chemistry file-naming note:

For this submission, public supporting material should be uploaded using Frontiers-style Supplementary Data Sheet numbering. The files prepared in this package correspond to Supplementary Data Sheets 1-6 and Supplementary Video 1.

Purpose:

This README/data dictionary documents the supplementary material accompanying the S-Remiketamine molecular-modeling manuscript. It explains the file contents, compound naming conventions, units, abbreviations, figure source-data mapping, and interpretation limits. It is intended to support transparency, reproducibility, and reviewer assessment of the computational workflow.

Important interpretation note:

All docking scores, molecular-dynamics outputs, MM-GBSA estimates, CES1 geometric descriptors, ADMET predictions, and metabolite-prediction outputs are computational, comparative, and hypothesis-generating. They should not be interpreted as experimental receptor affinity, receptor potency, hydrolysis rate, enzymatic turnover, plasma clearance, metabolite formation in vivo, pharmacokinetics, clinical efficacy, or clinical safety.

Additional source-audit note:

The revised Frontiers in Chemistry-adapted package was checked against uploaded method documentation, docking reports, MD reports, trajectory analyses, quantitative geometry files, SDF files, SMILES files, and figure source data. SDF-derived formulae and exact masses are treated as the final compound-identity source where older intermediate workbooks differ. In particular, R14 is reported as $C_{17}H_{20}ClNO_3$ with exact MW 321.113171 Da, and R11 is reported as $C_{15}H_{18}ClNO_3$ with exact MW 295.097521 Da.

1. Intended public file inventory

File	File type	Content / use
Manuscript_S_Remiketamine.docx or final checked manuscript file	Main manuscript	Main manuscript containing the title, abstract, introduction, computational methods, results, discussion, limitations, conclusion, declarations, data availability, author contributions, and references.
Graphical_Abstract.tiff or Graphical_Abstract.png	Graphical abstract / manuscript graphic	Graphical summary of the workflow from S-ketamine to S-Remiketamine, the R1-R20 analogue library, and the integrated computationally prioritized analogue.
Figure_1	Main figure	Chemical-structure figure showing S-ketamine, S-Remiketamine, R2, R11, and R14. No numerical source-data CSV is required for this structural illustration.
Figure_2	Main figure	Ranked NMDA docking-score summary across representative analogues; numerical values are provided in Supplementary Data Sheet 3.
Figure_3	Main figure	Cross-target docking heat map across NMDA receptor and off-target panels; numerical values are provided in Supplementary Data Sheet 3 and Supplementary Table S2.
Figure_4	Main figure	NMDA receptor molecular-dynamics snapshots and relative NMDA MM-

		GBSA support for R2, R11, and R14; numerical values are provided in Supplementary Data Sheet 3 and Supplementary Table S3.
Figure_5	Main figure	CES1 molecular-dynamics snapshots and CES1 MM-GBSA comparison at 0 ns and 100 ns for R2, R11, and R14; numerical values are provided in Supplementary Data Sheet 3 and Supplementary Table S4.
Figure_6	Main figure	Integrated normalized comparative performance plot for R2, R11, and R14; numerical values are provided in Supplementary Data Sheet 3.
Supplementary_Data_Sheet_1_Supplementary_Methods.docx	Supplementary Data Sheet	Conservative supplementary methods describing ligand preparation, protein preparation, docking, molecular dynamics, MM-GBSA analysis, CES1 geometric assessment, ADMET prediction, metabolite prediction, source-data generation, and interpretation limits.
Supplementary_Data_Sheet_2_Supplementary_Tables.zip	Supplementary Data Sheet ZIP containing DOCX tables	Compound identity and structural descriptor table for S-ketamine, S-Remiketamine, and R1-R20.
Supplementary_Data_Sheet_2_Supplementary_Tables.zip	Supplementary Data Sheet	Complete comparative docking-score matrix across NMDA receptor and off-target panels, including reference ligands and derived summary metrics.
Supplementary_Data_Sheet_2_Supplementary_Tables.zip	Supplementary Data Sheet	Dynamic NMDA and CES1 comparative summaries for R2, R11, and R14, including MM-GBSA and CES1 geometric descriptors.
Supplementary_Data_Sheet_2_Supplementary_Tables.zip	Supplementary Data Sheet	Predictive ADMET and metabolite-prediction summary for the prioritized compounds.
Supplementary_Data_Sheet_2_Supplementary_Tables.zip	Supplementary Data Sheet	Condensed reader-orientation tables summarizing structural rationale, representative docking trends, and integrated R2/R11/R14 computational prioritization. These tables do not introduce additional experimental data.
Supplementary_Data_Sheet_3_Figure_Source_Data.csv	Source data	Merged numerical source-data file for Figures 2-6 in tidy long format.
Supplementary_Data_Sheet_4_Isomeric_SMILES.csv	Machine-readable structure data	Isomeric SMILES and compound identifiers for S-ketamine, S-Remiketamine, and R1-R20. Compound identity should be interpreted together with Supplementary Table S1 and the SDF structure archive.
Supplementary_Data_Sheet_5_SDF_Structure_Files.zip	Machine-readable structure archive	SDF files for S-ketamine, S-Remiketamine, and R1-R20.
Supplementary_Data_Sheet_6_README_Data_Dictionary.pdf	README / data dictionary	This file. Provides file descriptions, terminology, units, abbreviations, source-data mapping, and interpretation limits.

Note:

Native computational job records, including LigPrep/Epik, Glide, Desmond, Prime MM-GBSA, trajectory-analysis, SwissADME, BioTransformer, and related source records, may be provided separately for editorial or reviewer inspection where permitted by the submission system. Such native job records are distinct from the public supplementary material listed above.

2. Compound naming conventions

Term	Role	Definition / use in this manuscript package
S-ketamine	Parent comparator	Reference S-configured arylcyclohexanone scaffold used to benchmark N-substitution effects. Use "S-ketamine" where stereochemistry matters.
S-Remiketamine	Novel prototype scaffold	S-ketamine-derived N-substituted prototype scaffold and conceptual anchor for the R-series analogue library. Use "S-Remiketamine" consistently.
R1-R20	Second-stage analogue library	Systematically varied S-Remiketamine-derived analogues exploring ester, carbamate, acyl, oxo-linked, dicarbonyl-containing, and hybrid N-substituent motifs.
R2	Static docking/NMDA-preference benchmark	Strongest static NMDA docking/NMDA-preference benchmark in the reported comparative workflow. R2 should not be described as the integrated dynamic/CES1 candidate.
R11	Mechanistic comparator	Polar glycolyl-linker comparator used to understand how a more polar

		linker architecture affects NMDA and CES1-oriented analyses.
R14	Integrated computationally prioritized analogue	Prioritized analogue when NMDA dynamic stability, reduced predicted off-target compatibility, CES1 active-site-compatible structural behavior, MM-GBSA support, and predictive ADMET/metabolite data are considered together. This is not an experimentally validated pharmacological lead.
S-Remi	Deprecated / avoid	Short label formerly used in earlier draft figures. The preferred label for this submission package is "S-Remiketamine."

Source-of-truth convention:

For compound identity, the SDF source structures, the SMILES file, and Supplementary Table S1 should be interpreted together. Matching molecular formula alone should not be treated as proof of structural identity because constitutional, linker-level, or substituent-connectivity differences may remain.

3. Data dictionary for Supplementary Data Sheet 3

Supplementary Data Sheet 3 contains the numerical source data used to generate Figures 2-6. It is organized in tidy long format.

Column	Type	Meaning	Unit
Supplementary_Data_File	Text	Name or identifier of the merged source-data file.	None
Figure	Text	Figure number associated with the row.	None
Figure_Title	Text	Manuscript figure title or short figure description.	None
Source_CSV	Text	Original individual source-data CSV from which the row was derived, if applicable.	None
Original_Row_Number	Integer	Row number in the original source-data CSV, if retained.	None
Compound	Text	Compound identifier, for example S-ketamine, S-Remiketamine, R2, R11, or R14.	None
Original_Metric_Name	Text	Original metric name retained from the source-data file.	None
Metric_Label	Text	Reader-friendly label for the metric.	None
Numeric_Value	Numeric	Numeric value used in the figure. Blank if the entry is textual.	Metric-specific
Text_Value	Text	Textual value or interpretive label. Blank if the source entry is numeric.	None
Unit	Text	Unit applicable to Numeric_Value.	See unit dictionary below

4. Unit dictionary

Unit	Used for	Interpretation
kcal/mol	Glide docking scores and MM-GBSA energy terms.	Used comparatively within the same computational workflow. More negative docking scores indicate stronger predicted binding only within a given target-specific docking model.
Da	Molecular weight / exact molecular weight.	Daltons.
Angstrom	Distance values in geometric analyses.	Used for ligand-receptor or ligand-enzyme distances, including distances between ligand carbonyl centers and catalytic/proximal CES1 residues.
Angstrom squared	Topological polar surface area.	Used for TPSA.
Degrees	Attack-angle or geometric-angle measurements.	Used in CES1 geometric summaries.
%	Occupancy values.	Percentage of frames or events meeting the reported interaction criterion within the analyzed trajectory summary.
Unitless	LogP, pKa, formal charge, normalized relative performance, and preference-derived metrics.	These values have no physical unit; normalized performance values are scaled within the relevant comparison set.

5. Key abbreviations

Abbreviation	Definition
ADMET	Absorption, distribution, metabolism, excretion, and toxicity
BBB	Blood-brain barrier
BOILED-Egg	SwissADME-style graphical prediction model for gastrointestinal absorption and brain penetration
CES1	Human carboxylesterase 1
CYP	Cytochrome P450 enzyme family
DAT	Dopamine transporter
GI	Gastrointestinal
HBA / HBD	Hydrogen-bond acceptor / hydrogen-bond donor
hERG	Human ether-a-go-go-related gene potassium channel
KOR / kappa / kappa-opioid receptor	Kappa-opioid receptor
MD	Molecular dynamics
MM-GBSA	Molecular mechanics-generalized Born surface area
MOR / mu / mu-opioid receptor	Mu-opioid receptor
MW	Molecular weight
NMDA	N-methyl-D-aspartate
P-gp	P-glycoprotein
PDB	Protein Data Bank
RMSD	Root-mean-square deviation
RMSF	Root-mean-square fluctuation
SDF	Structure-data file
SERT	Serotonin transporter
SMILES	Simplified Molecular Input Line Entry System
sigma-1 / sigma1R	Sigma-1 receptor
TPSA	Topological polar surface area
UGT	UDP-glucuronosyltransferase

6. Figure-specific source-data mapping

Figure	Source-data location	Data represented
Figure 1	Structural figure; SDF and SMILES files	Representative chemical structures of S-ketamine, S-Remiketamine, R2, R11, and R14. No numerical CSV source data are required for this structural illustration.
Figure 2	Supplementary Data Sheet 3	Ranked NMDA docking scores for representative analogues; unit: kcal/mol.
Figure 3	Supplementary Data Sheet 3 and Supplementary Table S2	Cross-target docking scores across NMDA, SERT, DAT, mu-opioid receptor, kappa-opioid receptor, sigma-1 receptor, and hERG; unit: kcal/mol.
Figure 4	Supplementary Data Sheet 3 and Supplementary Table S3	Relative NMDA MM-GBSA support for R2, R11, and R14; unit: kcal/mol.
Figure 5	Supplementary Data Sheet 3 and Supplementary Table S4	CES1 MM-GBSA values at 0 ns and 100 ns for R2, R11, and R14; unit: kcal/mol.
Figure 6	Supplementary Data Sheet 3	Normalized integrated performance values for NMDA docking, internal mean off-target preference, and CES1 stabilization; unitless.

7. Structural data files

Supplementary Data Sheet 4: Isomeric SMILES file

The SMILES file contains isomeric SMILES strings and compound identifiers for S-ketamine, S-Remiketamine, and R1-R20. The file should be used with Supplementary Table S1 and the SDF archive to confirm structural identity.

Supplementary Data Sheet 5: SDF archive

The SDF archive contains machine-readable structure files for the parent comparator, prototype scaffold, and R-series analogues. SDF records are provided to support structural traceability and import into molecular-modeling or cheminformatics software.

Structural interpretation guardrail:

If any discrepancy is identified among a figure, table, SMILES entry, or SDF file, the final curated SDF structure archive and Supplementary Table S1 should be treated as the primary compound-identity references, and the discrepancy should be resolved before reuse.

8. Interpretation guardrails

1. Docking scores are computational ranking metrics, not experimental binding affinities.
2. More negative docking scores indicate stronger predicted binding only within a target-specific docking model and should not be compared as absolute affinities across unrelated proteins.
3. Mean Delta(off-target - NMDA) is an internal NMDA-preference/off-target-compatibility metric calculated as the average of off-target docking score minus NMDA docking score across the off-target panel. Larger positive values indicate weaker predicted mean off-target binding relative to NMDA within this internal computational framework.
4. Molecular-dynamics and MM-GBSA analyses provide comparative support for relative complex stability and structural compatibility under the stated modeling assumptions. They do not measure receptor potency, receptor gating, channel conductance, residence time, catalytic rate, or clinical activity.
5. NMDA molecular-dynamics simulations are interpreted as comparative explicit-solvent simulations and should not be treated as full physiological models of ion-channel gating.
6. CES1 docking, molecular dynamics, MM-GBSA, and geometric analyses support structural compatibility with esterase engagement. They do not establish hydrolysis rate, enzymatic turnover, plasma stability, plasma clearance, or in vivo pharmacokinetics.
7. ADMET and metabolite-prediction outputs are predictive. They do not experimentally validate BBB penetration, metabolic pathway dominance, metabolite formation, metabolite inactivity, excretion route, pharmacokinetic behavior, toxicology, or safety.
8. The manuscript and supplementary material are intended to guide future synthesis and experimental validation. Experimental receptor pharmacology, enzymology, plasma-stability testing, metabolite verification, and PK/PD studies are required before biological or clinical conclusions can be drawn.

9. Data and software availability notes

Curated source data supporting the manuscript are provided in the supplementary material listed above. Commercial molecular-modeling workflows used for ligand preparation, receptor preparation, docking, molecular dynamics, and MM-GBSA analysis were performed using Schrödinger-style tools as described in the manuscript and Supplementary Methods. Predictive ADMET and metabolite-route assessments used SwissADME-style and BioTransformer-style outputs as described in the manuscript and Supplementary Methods. No new custom software or algorithm is reported in this study.

Where native job files, software build labels, run-level configuration files, or trajectory-analysis exports are required for audit-level reproducibility, these records may be provided separately for editorial or reviewer inspection where permitted by the submission system.
