# Supplementary material for: Structure-based molecular modeling of a novel S-Remiketamine scaffold and derived arylcyclohexanone analogues: NMDA receptor dynamics, MM-GBSA ranking, and CES1-Oriented structural compatibility
Source: Front Chem. 2026 Sep 3;14:1922077. doi: 10.3389/fchem.2026.1922077 (PMC13582596; doi:10.3389/fchem.2026.1922077)
Supplement: Supplementary file 2 [file Supplementaryfile1.docx]

# Supplementary Data Sheet 1: Supplementary Methods

Supplementary material for Frontiers in Chemistry. This file corresponds to Supplementary Data Sheet 1 in the submission package.

## 1. Purpose and interpretation limits

This Supplementary Methods file provides conservative methodological detail for the computational workflow used in the S-Remiketamine structure-based molecular-modeling and computational medicinal-chemistry manuscript. It supports transparent interpretation of ligand preparation, receptor docking, molecular dynamics, MM-GBSA analyses, CES1 active-site geometric assessment, ADMET prediction, metabolite prediction, and associated source-data files. Additional source folders supplied for audit included method documentation, docking/comparative reports, MD simulation reports, trajectory analyses, quantitative geometry analyses, SDF/SMILES records, and figure/source-data files. Where intermediate source documents differed, the final curated SDF structures, Supplementary Tables, numerical source-data files, and manuscript-ready summaries were treated as the source of truth.

All outputs are computational, comparative, and hypothesis-generating. Docking scores are internal target-specific prioritization metrics and are not experimental binding affinities. MD and MM-GBSA analyses provide comparative stability and structural-compatibility information under the modeling assumptions described below. CES1 docking, MD, MM-GBSA, and geometric analyses support structural compatibility with esterase engagement but do not measure hydrolysis rate, enzymatic turnover, plasma clearance, or in vivo pharmacokinetics. ADMET and metabolite-prediction outputs are predictive and should not be interpreted as experimental pharmacokinetic, toxicological, or functional validation.

## 2. Compound naming conventions and ligand set

The ligand series follows a two-stage design pathway. S-ketamine is the parent comparator. S-Remiketamine is the novel prototype scaffold derived from S-ketamine by rational N-substitution. R1-R20 are second-stage analogues derived from the S-Remiketamine design concept to explore controlled variation in substituent class, steric bulk, polarity, linker chemistry, hydrogen-bonding capacity, and predicted CES1 active-site compatibility. The claim of novelty is restricted to the specific S-Remiketamine scaffold and R1-R20 library described in this submission. The design rationale is soft-drug-inspired and ester-enabled, conceptually comparable to established short-acting anesthetic strategies such as remifentanil and remimazolam; however, no experimental equivalence, receptor potency, CES1 hydrolysis, pharmacokinetics, efficacy, or safety is claimed in this supplementary file.

| **Compound/group** | **Role in study** | **Naming convention** |
| --- | --- | --- |
| S-ketamine | Parent comparator and reference arylcyclohexanone scaffold | Use S-ketamine where stereochemistry matters. |
| S-Remiketamine | Novel prototype scaffold and conceptual anchor for the optimization library | Use S-Remiketamine consistently. |
| R1-R20 | Second-stage analogue library derived from the S-Remiketamine concept | Use R-series identifiers exactly as in Supplementary Table S1 and source structure files. |
| R2 | Strongest static NMDA docking/NMDA-preference benchmark | Do not describe R2 as the integrated dynamic/CES1 candidate. |
| R11 | Mechanistic polar-linker comparator | Describe R11 as a comparator rather than a prioritized candidate. |
| R14 | Prioritized integrated computational candidate | Prioritized by combined NMDA dynamic support, reduced predicted off-target compatibility, and CES1 active-site-oriented structural behavior. |

## 3. Computational workflow overview

- Structure curation and ligand preparation for S-ketamine, S-Remiketamine, and R1-R20.
- Protein preparation and receptor-grid generation for NMDA receptor, off-target proteins, and CES1.
- Comparative molecular docking of the compound set across NMDA and off-target panels.
- Selection of R2, R11, and R14 for dynamic analyses based on docking, NMDA-preference/off-target-compatibility, and design-role considerations.
- Explicit-solvent MD simulations for selected NMDA and CES1 complexes under matched comparative conditions.
- MM-GBSA and CES1 geometric analyses to support relative stability and CES1 active-site-compatibility interpretation.
- In silico ADMET and metabolite prediction used only as predictive, hypothesis-generating support.

## 4. Ligand preparation

Three-dimensional ligand structures were prepared for S-ketamine, S-Remiketamine, and R1-R20. Preparation preserved the intended S-configuration at the stereogenic center and generated low-energy structures suitable for docking and simulation. Available ligand-preparation logs in the supplied source archive include Schrödinger LigPrep records, including LigPrep 2022-4 build 134 examples with Epik-based enumeration, desalting/neutralization, stereo handling, and geometry optimization. The final manuscript does not rely on all enumerated states; compound identity is defined by the curated final SDF/SMILES structures and Supplementary Table S1. SDF-derived formulae and exact masses were used to resolve descriptor discrepancies, including R11 (C15H18ClNO3; exact MW 295.097521 Da) and R14 (C17H20ClNO3; exact MW 321.113171 Da).

## 5. Protein targets and receptor preparation

The workflow used the NMDA receptor as the primary pharmacological target, a panel of off-target proteins for off-target-compatibility assessment, and human CES1 as an CES1 active-site-compatibility model. Protein preparation used standard Schrödinger-style steps, including bond-order assignment, hydrogen addition, protonation-state optimization, hydrogen-bond network optimization, local minimization, and grid generation around co-crystallized ligands or defined active-site regions. Source documentation identifies the main structures as NMDA receptor PDB 7EU8, SERT PDB 5I6X, DAT PDB 9EO4, mu-opioid receptor PDB 5C1M, kappa-opioid receptor PDB 9D61, sigma-1 receptor PDB 6DJZ, hERG PDB 5VA1, and CES1 PDB 1MX9 in the final manuscript package. Docking and NMDA-preference/off-target-compatibility interpretations were made comparatively within each target model and not as absolute affinity estimates.

## 6. Molecular docking and NMDA-preference metrics

Docking was performed using Glide-style receptor docking. The full ligand set was docked to NMDA and the off-target panel, and selected CES1 docking reports evaluated the proximity and orientation of labile carbonyl-containing substituents relative to the esterase active-site environment. Available source reports describe Glide XP-style docking, receptor-grid generation around relevant active-site or co-crystallized ligand regions, and post-docking inspection of hydrogen-bond, hydrophobic, and geometric interaction patterns. CES1 docking source reports specifically monitored Ser221 O-gamma to ligand-carbonyl geometry and oxyanion-hole contacts involving Gly142/Gly143 and Ala222 where available. Docking scores were interpreted comparatively within the same target-specific model and were not treated as absolute binding free energies or experimental affinities. Mean Delta(off-target - NMDA) was calculated as average(off-target docking score - NMDA docking score) across SERT, DAT, mu-opioid, kappa-opioid, sigma-1, and hERG models.

## 7. Selection of compounds for dynamic analysis

R2, R11, and R14 were selected for deeper dynamic evaluation because they represent distinct roles in the design space: R2 as the strongest static docking/NMDA-preference benchmark, R11 as a polar-linker mechanistic comparator, and R14 as the integrated computationally prioritized candidate with competitive NMDA docking, reduced predicted off-target compatibility, and favorable CES1 active-site-oriented structural behavior.

## 8. NMDA receptor molecular-dynamics simulations

NMDA receptor-ligand complexes for R2, R11, and R14 were simulated in explicit solvent under matched comparative conditions. The primary submitted comparative NMDA simulations were 100 ns simulations performed without an explicit membrane bilayer and are therefore interpreted as relative comparisons of dynamic stability, contact persistence, and conformational restriction rather than as full physiological reconstructions of channel gating or ion conductance. Source-methodology records describe Desmond-style explicit-solvent simulations using orthorhombic periodic boxes with at least a 10 Å solvent buffer, neutralization with counter-ions and 0.15 M NaCl, NPT production dynamics, a 2 fs integration timestep, 9.0 Å short-range cutoffs, Particle Mesh Ewald electrostatics, and bond constraints involving hydrogen atoms. Trajectory analyses included RMSD, RMSF, secondary-structure elements where available, ligand-protein contact persistence, hydrogen-bond occupancy, water-mediated interactions, and residue-level interaction summaries. A separate source report describing a membrane-context R14 simulation was reviewed but was not used for the primary R2/R11/R14 comparative ranking because matched membrane-context simulations were not available for all three advanced compounds.

## 9. CES1 molecular-dynamics and geometric analyses

Docked CES1 complexes of R2, R11, and R14 were subjected to explicit-solvent MD under matched comparative conditions. CES1 analyses assessed ligand stability in the esterase pocket, persistence of structurally compatible orientations, and relative behavior near the Ser221-centered catalytic environment. These analyses are structural and comparative; they do not measure hydrolysis rate or enzymatic turnover. The canonical residue-numbering convention used in the manuscript maps internal Maestro/Desmond residue labels to standard CES1 active-site notation, including Ser221, Gly142/Gly143, Ala222, His468, and Glu354. Internal Maestro/Desmond residue-label differences are retained only in native source records. Final tables use canonical residue labels for clarity and reader accessibility.

## 10. MM-GBSA calculations

Relative complex stability was estimated using Prime MM-GBSA-style calculations on equilibrated or selected trajectory frames for both NMDA and CES1 systems. Reported values are comparative support for relative stability, not direct experimental measures of receptor potency, inhibitor residence time, catalytic rate, hydrolysis rate, or pharmacokinetic behavior. The submitted summary tables retain only values explicitly available in the curated source set; older interpretive phrases suggesting proven rapid hydrolysis, enzymatic turnover, or receptor potency were deliberately excluded from the submission-ready manuscript.

## 11. ADMET and metabolite-prediction workflow

Selected prioritized compounds were evaluated using in silico ADMET and metabolite-prediction tools. SwissADME-style descriptors included molecular weight, topological polar surface area, lipophilicity, hydrogen-bond donors/acceptors, qualitative gastrointestinal absorption, BBB/BOILED-Egg interpretation, P-glycoprotein prediction, and CYP prediction flags where available. BioTransformer-style outputs were used to summarize explicitly recoverable predicted metabolite routes. These predictions do not validate in vivo pharmacokinetics, safety, efficacy, or metabolite formation.

## 12. Methodological limitations

No experimental receptor-binding, functional electrophysiology, enzymology, plasma hydrolysis, metabolite-identification, toxicology, or PK/PD data are contained in this supplementary methods file. Docking rankings are target-model-specific. The primary comparative NMDA MD simulations lack an explicit membrane bilayer, and the separate membrane-context R14 source report is not used as a matched comparative dataset. CES1 structural compatibility does not establish hydrolysis rate or catalytic turnover without dedicated enzymology or reaction-coordinate methods. Predictive ADMET and metabolite outputs support prioritization only. Any apparent discrepancy among intermediate workbooks, narrative reports, and structure files was resolved in favor of the final curated SDF/SMILES structures and Supplementary Tables used in the manuscript.

**13. Reproducibility and data traceability**

The public source package includes compound identity and structure files, complete docking matrices, dynamic NMDA/CES1 summaries, figure-level numerical source data, and a README/data dictionary. Compound identity should be interpreted from Supplementary Table S1 together with the isomeric SMILES file and SDF structure archive. PDB identifiers and target roles are reported in the main manuscript. Native job-level computational records, including software builds and run-level configuration files where available, are retained for editorial or reviewer inspection where permitted by the submission system. No wet-laboratory, receptor-binding, electrophysiology, enzymology, plasma-stability, metabolite-identification, or PK/PD data are claimed in this supplementary methods file.
