# Supplementary material for: Structure-based molecular modeling of a novel S-Remiketamine scaffold and derived arylcyclohexanone analogues: NMDA receptor dynamics, MM-GBSA ranking, and CES1-Oriented structural compatibility
Source: Front Chem. 2026 Sep 3;14:1922077. doi: 10.3389/fchem.2026.1922077 (PMC13582596; doi:10.3389/fchem.2026.1922077)
Supplement: Supplementary file 3 [file DataSheet1.zip › Supplementary_Data_Sheet_2_Supplementary_Tables/Supplementary Tables_S3_S4_Dynamic_NMDA_CES1_summaries.docx]

**Supplementary Tables S3–S4**
*Dynamic NMDA- and CES1-Oriented Comparative Summary for R2, R11, and R14*

**Table note.** This supplementary file curates the dynamic-analysis results into submission-oriented summary tables for the NMDA receptor and CES1 systems. Quantitative entries are limited to values explicitly available in the final project source set. Interpretations are restricted to conservative comparative conclusions aligned with the final manuscript and should not be read as direct experimental evidence of receptor potency, hydrolysis rate, enzymatic turnover, plasma clearance, or in vivo pharmacokinetics.

**Source-prioritization note.** For Table S4B, the structured CES1 geometry workbook was used as the primary source because it provided internally consistent mean ± SD values for all three compounds. Narrative descriptions were used only for contextual interpretation and were not allowed to override the workbook-derived quantitative values.

**Table S3A. Comparative NMDA receptor MM-GBSA summary (100 ns end-state values)**

Values are reported for the prioritized compounds examined in detail by NMDA receptor molecular dynamics. Docking scores are included to maintain direct linkage with the main-manuscript prioritization framework.

| **Compound** | **NMDA docking score** | **ΔGbind total (kcal/mol)** | **ΔGCoulomb** | **ΔGCovalent** | **ΔGHbond** | **ΔGLipo** | **ΔGvdW** | **Ligand efficiency** |
| --- | --- | --- | --- | --- | --- | --- | --- | --- |
| R2 | -7.308 | -41.53 | -7.14 | +4.04 | -0.42 | -22.45 | -17.88 | -1.89 |
| R11 | -4.681 | -28.80 | -2.64 | +6.22 | -0.78 | -20.73 | -14.12 | -1.44 |
| R14 | -6.476 | -42.10 | -6.16 | +2.86 | -0.56 | -25.87 | -18.44 | -1.91 |

Interpretive note: within the uploaded NMDA dynamic dataset, R14 retained the most favorable integrated end-state MM-GBSA support, whereas R2 remained the strongest static docking benchmark and R11 served primarily as a mechanistic polar-linker comparator.

**Table S3B. Key NMDA receptor interactions maintained during MD simulation**

Occupancy values below are taken from the 100 ns interaction summaries and are best interpreted comparatively rather than as absolute biophysical measurements.

| **Compound** | **Residue / bridge** | **Interaction type** | **Reported occupancy** | **Conservative structural significance** |
| --- | --- | --- | --- | --- |
| R2 | Asn616 | Direct H-bond | 87% | Primary polar anchor in NMDA pocket |
| R2 | Asn615 | Direct H-bond | 68% | Secondary polar stabilization |
| R2 | Val644 | Hydrophobic / CH-pi | 72% | Lipophilic pocket anchoring |
| R2 | Ala645 | Water-mediated H-bond | 54% | Bridged stabilization of terminal group |
| R2 | Thr648 | H-bond | 60% | Dynamic polar capping near vestibule |
| R2 | Leu643 | Hydrophobic | 79% | Supports pocket packing |
| R2 | Asn616-Thr648 | Water bridge | ~40% | Persistent hydration shell |
| R11 | Asn616 | Direct H-bond | 83% | Highly persistent carbonyl alignment |
| R11 | Asn615 | Direct H-bond | 64% | Polar stabilization with solvent exchange |
| R11 | Val644 | Hydrophobic | 69% | Pocket anchoring |
| R11 | Ala645 | Water-mediated H-bond | 51% | Bridged stabilization of linker |
| R11 | Thr648 | H-bond | 57% | Polar capping of ligand tail |
| R11 | Asn616-Ala645 | Water bridge | ~35% | Orientation flexibility |
| R14 | Asn616 | Direct H-bond | 91% | Most stable direct H-bond of the set |
| R14 | Asn615 | Direct H-bond | 74% | Reinforces polar anchoring |
| R14 | Val644 | Hydrophobic | 70% | Stable nonpolar support |
| R14 | Ala645 | Water-mediated H-bond | 63% | Orients acetoacetyl dicarbonyl functionality |
| R14 | Thr648 | H-bond | 66% | Persistent polar contact |
| R14 | Asn616-Ala645 | Water bridge | ~48% | Most frequent bridging-water interaction |

**Table S4A. Comparative CES1 MM-GBSA summary**

For CES1, both the initial docked state (0 ns) and the 100 ns end-state values are retained because the direction of MM-GBSA change was central to the final manuscript interpretation.

| **Compound** | **ΔGbind (0 ns)** | **ΔGbind (100 ns)** | **ΔΔG (change)** | **ΔGCoulomb (100 ns)** | **ΔGvdW (100 ns)** | **ΔGLipo (100 ns)** | **ΔGsolv (100 ns)** | **Ligand efficiency (100 ns)** |
| --- | --- | --- | --- | --- | --- | --- | --- | --- |
| R2 | -91.36 | -81.04 | +10.32 | -1.94 | -25.94 | -45.38 | +26.18 | -1.87 |
| R11 | -97.28 | -84.28 | +13.00 | -5.53 | -28.04 | -49.30 | +23.45 | -2.00 |
| R14 | -83.91 | -99.44 | -15.53 | -23.51 | -28.62 | -51.22 | +24.18 | -2.44 |

Interpretive note: R14 was the only prioritized compound in this set whose CES1 MM-GBSA became more favorable over simulation time, whereas R2 and R11 both became less favorable despite retaining structurally compatible orientations in the CES1 active-site model.

**Table S4B. CES1 active-site geometry summary from the uploaded geometry workbook**

These values are reported from the structured workbook for R2, R11, and R14. They were retained as the primary quantitative source for geometric comparison because the workbook offered direct mean ± SD values for all three compounds.

| **Compound** | **Ser221 Oγ-carbonyl C distance (Å)** | **Attack angle (°)** | **Catalytic-triad H-bond occupancy (%)** | **Oxyanion-hole H-bond occupancy (%)** | **Water-bridge occupancy (%)** | **Conservative integrated interpretation** |
| --- | --- | --- | --- | --- | --- | --- |
| R2 | 2.82 ± 0.14 | 102.4 ± 2.6 | 84.3 ± 4.5 | 78.2 ± 3.9 | 42.5 ± 5.2 | CES1 active-site-compatible geometry with lower water-bridge occupancy and less favorable MM-GBSA evolution. |
| R11 | 2.73 ± 0.11 | 104.8 ± 2.3 | 91.7 ± 3.2 | 85.4 ± 2.8 | 57.9 ± 4.1 | Strong modeled catalytic-triad and oxyanion-hole engagement, but MM-GBSA became less favorable over the simulation. |
| R14 | 2.75 ± 0.09 | 106.1 ± 1.9 | 93.2 ± 2.6 | 88.5 ± 2.1 | 61.7 ± 3.8 | Most favorable integrated CES1 structural profile: strongest retained interaction network and improved MM-GBSA over time. |

**Interpretation notes:**

The values reported in Supplementary Tables S3–S4 are limited to directly extractable comparative docking, MD, MM-GBSA, and geometric parameters available in the final project source set. Descriptive terms implying measured hydrolysis rate, enzymatic turnover, receptor potency, plasma clearance, or in vivo pharmacokinetics are not used unless directly supported by quantitative experimental data. Accordingly, these tables should be interpreted as comparative computational summaries rather than as evidence of experimentally measured kinetic behavior.
