# Supplementary material for: Structure-based molecular modeling of a novel S-Remiketamine scaffold and derived arylcyclohexanone analogues: NMDA receptor dynamics, MM-GBSA ranking, and CES1-Oriented structural compatibility
Source: Front Chem. 2026 Sep 3;14:1922077. doi: 10.3389/fchem.2026.1922077 (PMC13582596; doi:10.3389/fchem.2026.1922077)
Supplement: Supplementary file 3 [file DataSheet1.zip › Supplementary_Data_Sheet_2_Supplementary_Tables/Supplementary Tables_S6_S8_Reader_orientation_summaries.docx]

**Supplementary Tables S6-S8. Condensed reader-orientation summary tables**

Supplementary Tables S6-S8 are provided as condensed reader-orientation summaries. Full compound-identity data, complete docking matrices, dynamic NMDA summaries, CES1 active-site geometric analyses, and ADMET/metabolite-prediction summaries are provided in Supplementary Tables S1-S5. These summary tables do not introduce additional experimental data and should be interpreted within the computational limitations stated in the manuscript.

**Supplementary Table S6. Condensed structural classes and design rationale for representative S-configured arylcyclohexanone analogues**

| **Compound** | **N-substituent class** | **Design intent** | **Advanced analysis** |
| --- | --- | --- | --- |
| S-ketamine | Parent arylcyclohexanone | Reference NMDA-antagonist scaffold and comparator for N-substitution effects | No |
| S-Remiketamine | Acyloxymethyl ester prototype | Novel prototype soft-drug-inspired scaffold introducing a predicted CES1 active-site-oriented N-substituent | No |
| R1 | Carbamate analogue | Test whether dual donor/acceptor character improves NMDA vestibular engagement while reducing predicted off-target docking compatibility | No |
| R2 | Isobutyryl analogue | Probe hydrophobic filling of the NMDA vestibule with preserved predicted NMDA preference across off-target panels | Yes |
| R5 | Ethyl carbamate analogue | Evaluate balanced polarity and steric bulk within the carbamate subclass | No |
| R11 | Glycolyl linker analogue | Representative polar oxo-linker used as a mechanistic comparator for CES1 active-site/NMDA analyses | Yes |
| R14 | Acetoacetyl analogue | Representative dicarbonyl-containing candidate integrating NMDA stability with CES1 active-site structural compatibility | Yes |
| R20 | Acetoxy-carbamate hybrid | Test whether hybridization of ester and carbamate features preserves predicted NMDA preference while retaining predicted CES1 active-site structural compatibility | No |

**Supplementary Table S7. Condensed comparative docking-score summary for representative analogues across NMDA receptor and off-target panels**

Docking scores are reported in kcal/mol. More negative values indicate stronger predicted binding within a given target-specific model. These values are comparative computational metrics and are not experimental affinity measurements.

| **Compound** | **NMDA** | **SERT** | **DAT** | **mu** | **kappa** | **sigma-1** | **hERG** | **Priority note** |
| --- | --- | --- | --- | --- | --- | --- | --- | --- |
| S-ketamine | -5.981 | -4.334 | -4.313 | -5.086 | -5.353 | -4.568 | -2.322 | Parent comparator |
| S-Remiketamine | -6.448 | -3.604 | -3.244 | -3.051 | -3.379 | -2.486 | -1.038 | Novel prototype scaffold |
| R1 | -7.162 | -3.215 | -4.570 | -3.130 | -3.572 | -2.910 | -1.526 | High predicted NMDA preference |
| R2 | -7.308 | -3.023 | -3.641 | -3.160 | -3.663 | -4.041 | -1.104 | Top static docking/NMDA-preference benchmark |
| R5 | -6.949 | -3.955 | -4.396 | -2.458 | -3.310 | -3.683 | -3.506 | Strong carbamate-class comparator |
| R11 | -4.681 | -5.200 | -5.352 | -3.686 | -4.178 | -3.753 | -1.620 | Mechanistic comparator; weaker NMDA |
| R14 | -6.476 | -2.217 | -2.115 | -2.049 | -3.363 | -2.542 | -1.036 | Integrated computationally prioritized candidate |
| R20 | -6.949 | -3.955 | -4.396 | -2.548 | -3.310 | -3.683 | -3.506 | Hybrid comparator with favorable predicted NMDA preference |

**Supplementary Table S8. Condensed integrated comparison of R2, R11, and R14 across NMDA dynamics, CES1 active-site structural compatibility, and computational prioritization**

| **Feature** | **R2** | **R11** | **R14** |
| --- | --- | --- | --- |
| NMDA docking score | -7.308 | -4.681 | -6.476 |
| NMDA MD / MM-GBSA trend | Strongest static docking hit; dynamic NMDA stability remained favorable but slightly less favorable than R14 | Weakest NMDA stability of the three; useful as a polar-linker comparator | Most favorable integrated NMDA dynamic profile of the three |
| Representative NMDA MM-GBSA | -41.53 kcal/mol | -28.80 kcal/mol | -42.10 kcal/mol |
| CES1 MM-GBSA trend (0 -> 100 ns) | -91.36 -> -81.04 kcal/mol | -97.28 -> -84.28 kcal/mol | -83.91 -> -99.44 kcal/mol |
| CES1 active-site structural interpretation | CES1 active-site-compatible poses observed, but greater fluctuation and less convincing integrated structural profile | Strong modeled polar engagement, but more variable geometry over time | Most favorable modeled stabilization and structurally compatible CES1 active-site engagement |
| Off-target profile | Favorable internal NMDA-preference/off-target-compatibility profile across transporter, opioid, sigma-1, and hERG models | Limited internal NMDA-preference profile; useful as a polar-linker comparator | Reduced predicted off-target docking compatibility with preserved NMDA preference |
| Final role in study | Top static docking/NMDA-preference benchmark | Mechanistic comparator | Prioritized integrated computational candidate |
