# Supplementary material for: Structure-based molecular modeling of a novel S-Remiketamine scaffold and derived arylcyclohexanone analogues: NMDA receptor dynamics, MM-GBSA ranking, and CES1-Oriented structural compatibility
Source: Front Chem. 2026 Sep 3;14:1922077. doi: 10.3389/fchem.2026.1922077 (PMC13582596; doi:10.3389/fchem.2026.1922077)
Supplement: Supplementary file 3 [file DataSheet1.zip › Supplementary_Data_Sheet_2_Supplementary_Tables/Supplementary Table_S1_Compound_identity.docx]

**Supplementary Table S1. Full compound identity and structural descriptor table for S-ketamine, S-Remiketamine, and R1–R20**

**Table note.** Entries were compiled from the final project compound-identity records, source structural files, and manuscript source tables. All entries retain the S-configured arylcyclohexanone core unless otherwise stated. Where independently enumerated identifiers share the same molecular formula, this is flagged in the table; matching molecular formula alone should not be interpreted as proof of structural identity because constitutional, linker-level, or substituent-connectivity differences may remain.

| **Compound** | **Descriptor** | **N-substituent** | **R-group / linkage class** | **Molecular formula** | **Exact MW (Da)** | **Formal charge** | **Stereochemistry from SDF** | **Identity note** |
| --- | --- | --- | --- | --- | --- | --- | --- | --- |
| S-ketamine | Parent comparator | N–CH3 | Methyl | C13H16ClNO | 237.092042 | 0 | atom 3: S | Parent comparator scaffold |
| S-Remiketamine | Novel prototype scaffold | N–CH2–OCOCH3 | Acetoxy (ester) | C15H18ClNO3 | 295.097521 | 0 | atom 3: S | Formula duplicated with R11; duplication alone does not prove structural identity |
| R1 | Carbamate analogue | N–CH2–OCO–NHCH3 | Methyl carbamate | C15H19ClN2O3 | 310.108420 | 0 | atom 3: S | — |
| R2 | Isobutyryl analogue | N–CH2–OCOCH(CH3)2 | Isobutyryl | C17H22ClNO3 | 323.128821 | 0 | atom 3: S | — |
| R3 | Formyl analogue | N–CH2–OCHO | Formyl ester | C14H16ClNO3 | 281.081871 | 0 | atom 3: S | — |
| R4 | Chloroacetyl analogue | N–CH2–OCOCH2Cl | Chloroacetyl | C15H17Cl2NO3 | 329.058549 | 0 | atom 3: S | Structural duplicate/alias of R16 in the submitted SMILES/SDF source files |
| R5 | Ethyl carbamate analogue | N–CH2–OCO–NHC2H5 | Ethyl carbamate | C16H21ClN2O3 | 324.124070 | 0 | atom 3: S | Structural duplicate/alias of R20 in the submitted SMILES/SDF source files |
| R6 | Isopropyl chloroformate analogue | N–CH2–OCOOCH(CH3)2 | Isopropoxy carbonyl | C17H22ClNO4 | 339.123736 | 0 | atom 3: S | — |
| R7 | Hydroxymethylamine analogue | N–CH2OH | Hydroxymethyl | C13H16ClNO2 | 253.086956 | 0 | atom 3: S | Enumerated hydroxymethylamine analogue |
| R8 | Diacetyl analogue | N–CH2–OCOCH2–OCOCH3 | Diester chain | C17H20ClNO5 | 353.103000 | 0 | atom 3: S | — |
| R9 | Benzoyl analogue | N–CH2–OCOC6H5 | Benzoyl | C20H20ClNO3 | 357.113171 | 0 | atom 3: S | — |
| R10 | Chloroacetyl linker analogue | N–CH2–CO–CH2Cl | Oxo-linker with halogen substituent | C15H17Cl2NO2 | 313.063634 | 0 | atom 3: S | — |
| R11 | Glycolyl linker analogue | N–CH2–CO–CH2OH | Oxo-hydroxy linker | C15H18ClNO3 | 295.097521 | 0 | atom 3: S | Formula duplicated with S-Remiketamine; duplication alone does not prove structural identity |
| R12 | Oxalyl analogue | N–CO–COCH3 | Dicarbonyl linker | C15H16ClNO3 | 293.081871 | 0 | atom 3: S | — |
| R13 | Succinyl analogue | N–CH2–CO–CH2–COOH | Oxo-acid linker | C16H18ClNO4 | 323.092436 | 0 | atom 3: S | — |
| R14 | Acetoacetyl analogue | N–CH2–CO–CH2–COCH3 | β-diketone oxo-linker | C17H20ClNO3 | 321.113171 | 0 | atom 3: S | — |
| R15 | Oxamyl analogue | N–CO–NH–CH2–COOH | Amide-oxo linker | C15H17ClN2O4 | 324.087685 | 0 | atom 3: S | — |
| R16 | Chloroacetoxy analogue | N–CH2–O–CO–CH2Cl | Oxy–oxo hybrid linker | C15H17Cl2NO3 | 329.058549 | 0 | atom 3: S | Structural duplicate/alias of R4 in the submitted SMILES/SDF source files |
| R17 | Glycolyl carbamate analogue | N–CH2–O–CO–CH2OH–N–COCH3 | Mixed oxo–carbamate hybrid | C17H21ClN2O5 | 368.113899 | 0 | atom 3: S | — |
| R18 | Formyloxy analogue | N–CH2–O–CO–CHO | Simple oxo-ester | C15H16ClNO4 | 309.076786 | 0 | atom 3: S | — |
| R19 | Propionyl-oxo analogue | N–CH2–CO–CH2CH3 | Oxo-alkyl chain | C16H20ClNO2 | 293.118257 | 0 | atom 3: S | — |
| R20 | Acetoxy-carbamate hybrid | N–CH2–O–CO–NH–CH2CH3 | Dual ester–carbamate | C16H21ClN2O3 | 324.124070 | 0 | atom 3: S | Structural duplicate/alias of R5 in the submitted SMILES/SDF source files |

Abbreviations: MW, molecular weight.

**Source note:** N-substituent and R-group descriptors were compiled from the final project compound-identity records, source structural files, and manuscript source tables.
