# Supplementary material for: Structure-based molecular modeling of a novel S-Remiketamine scaffold and derived arylcyclohexanone analogues: NMDA receptor dynamics, MM-GBSA ranking, and CES1-Oriented structural compatibility
Source: Front Chem. 2026 Sep 3;14:1922077. doi: 10.3389/fchem.2026.1922077 (PMC13582596; doi:10.3389/fchem.2026.1922077)
Supplement: Supplementary file 3 [file DataSheet1.zip › Supplementary_Data_Sheet_2_Supplementary_Tables/Supplementary Table_S2_Docking_matrix.docx]

**Supplementary Table S2**

**Complete comparative docking-score table across NMDA receptor and off-target panels**

Scores are reported as Glide docking scores (kcal/mol). More negative values indicate stronger predicted binding within a given target-specific model. NMDA-preference/off-target-compatibility metrics should be interpreted comparatively within this computational workflow and not as experimental affinity or functional assay measurements.

*Off-target panels: SERT, DAT, μ-opioid receptor, κ-opioid receptor, sigma-1 receptor, and hERG. Mean off-target score and mean Δ(off-target − NMDA) are derived summary metrics calculated from the six off-target panels.*

**Table S2A. Reference ligands and benchmark docking scores**

| **Compound** | **NMDA** | **SERT** | **DAT** | **μ-Opioid** | **κ-Opioid** | **σ1** | **hERG** | **Role / comparator** |
| --- | --- | --- | --- | --- | --- | --- | --- | --- |
| MK-801 | -9.6 | — | — | — | — | — | — | NMDA benchmark |
| Paroxetine | — | −10.4 | — | — | — | — | — | SERT high-affinity reference |
| Fluoxetine | — | −9.8 | — | — | — | — | — | SERT reference |
| Cocaine | — | — | −11.45 | — | — | — | — | DAT reference |
| Morphine | — | — | — | −10.7 | −8.9 | — | — | Opioid agonist reference |
| Naloxone | — | — | — | −9.8 | −8.3 | — | — | Opioid antagonist reference |
| Haloperidol | — | — | — | — | — | −11.3 | — | Sigma-1 reference |
| Dofetilide | — | — | — | — | — | — | −11.7 | hERG reference |

**Table S2B. Test-compound comparative docking-score matrix and derived summary metrics**

| **Compound** | **Descriptor** | **NMDA** | **SERT** | **DAT** | **μ-Opioid** | **κ-Opioid** | **σ1** | **hERG** | **Mean off-target** | Mean Δ(off-target − NMDA) | **Interpretive note** |
| --- | --- | --- | --- | --- | --- | --- | --- | --- | --- | --- | --- |
| S-ketamine | Parent comparator | -5.981 | -4.334 | -4.313 | -5.086 | -5.353 | -4.568 | -2.322 | -4.329 | 1.652 | Moderate predicted NMDA preference |
| S-Remiketamine | Prototype scaffold | -6.448 | -3.604 | -3.244 | -3.051 | -3.379 | -2.486 | -1.038 | -2.800 | 3.648 | Predicted NMDA preference |
| R1 | Carbamate | -7.162 | -3.215 | -4.570 | -3.130 | -3.572 | -2.910 | -1.526 | -3.154 | 4.008 | Strong predicted NMDA preference |
| **R2** | Isobutyryl | -7.308 | -3.023 | -3.641 | -3.160 | -3.663 | -4.041 | -1.104 | -3.105 | 4.203 | Top static docking/NMDA-preference benchmark |
| R3 | Formyl | -5.023 | -3.884 | -4.258 | -2.786 | -3.128 | -2.279 | -3.170 | -3.251 | 1.772 | Weak predicted NMDA preference |
| R4 | Chloroacetyl | -5.933 | -4.015 | -4.218 | -1.971 | -3.019 | -3.557 | -2.770 | -3.258 | 2.675 | Moderate predicted NMDA preference |
| R5 | Ethyl carbamate | -6.949 | -3.955 | -4.396 | -2.458 | -3.310 | -3.683 | -3.506 | -3.551 | 3.398 | Strong predicted NMDA preference |
| R6 | Isopropyl chloroformate | -5.656 | -4.031 | -4.042 | -3.248 | -3.025 | -3.823 | -2.825 | -3.499 | 2.157 | Weak predicted NMDA preference |
| R7 | Hydroxymethylamine | -6.917 | -5.456 | -4.025 | -4.511 | -5.325 | -6.278 | -2.690 | -4.714 | 2.203 | Mixed predicted off-target profile |
| R8 | Diacetyl | -5.369 | -3.631 | -4.069 | -2.388 | -3.756 | -4.204 | -2.710 | -3.460 | 1.909 | Predicted NMDA preference |
| R9 | Benzoyl | -6.436 | -4.036 | -5.131 | -3.418 | -3.964 | -4.304 | -1.417 | -3.712 | 2.724 | Predicted NMDA preference |
| R10 | Chloroacetyl linker | -5.794 | -4.503 | -3.877 | -3.426 | -4.106 | -3.738 | -1.332 | -3.497 | 2.297 | Predicted NMDA preference |
| **R11** | Glycolyl linker | -4.681 | -5.200 | -5.352 | -3.686 | -4.178 | -3.753 | -1.620 | -3.965 | 0.716 | **Weak predicted NMDA preference** |
| R12 | Oxalyl | -5.874 | -5.774 | -4.622 | -5.173 | -5.623 | -3.031 | -3.140 | -4.561 | 1.313 | Moderate predicted NMDA preference |
| R13 | Succinyl | -5.380 | -4.487 | -4.674 | -4.213 | -5.499 | -3.999 | -2.847 | -4.287 | 1.093 | Broad weak predicted profile |
| **R14** | Acetoacetyl | -6.476 | -2.217 | -2.115 | -2.049 | -3.363 | -2.542 | -1.036 | -2.220 | 4.256 | **Highest internal NMDA-preference metric** |
| R15 | Oxamyl | -4.605 | -5.225 | -4.635 | -3.865 | -6.167 | -1.177 | -3.377 | -4.074 | 0.531 | Poor predicted NMDA preference |
| R16 | Chloroacetoxy | -5.993 | -4.015 | -5.084 | -1.971 | -3.019 | -3.557 | -2.770 | -3.403 | 2.590 | Predicted NMDA preference |
| R17 | Glycolyl carbamate | -5.610 | -4.306 | -4.513 | -5.610 | -4.355 | -4.077 | -2.435 | -4.216 | 1.394 | Broad weak predicted profile |
| R18 | Formyloxy | -5.230 | -4.983 | -4.664 | -3.779 | -6.467 | -5.374 | -2.856 | -4.687 | 0.543 | Off-target docking signal at κ/sigma-1 models |
| R19 | Propionyl-oxo | -6.402 | -4.555 | -4.132 | -3.980 | -5.192 | -3.683 | -2.169 | -3.952 | 2.450 | Predicted NMDA preference |
| R20 | Acetoxy-carbamate hybrid | -6.949 | -3.955 | -4.396 | -2.548 | -3.310 | -3.683 | -3.506 | -3.566 | 3.383 | Predicted NMDA preference |

**Abbreviations:** SERT, serotonin transporter; DAT, dopamine transporter; σ1, sigma-1 receptor; hERG, human ether-à-go-go-related gene potassium channel.

**Derived metric definition: Mean Δ(off-target − NMDA) = average of [off-target docking score − NMDA docking score] across SERT, DAT, μ-opioid, κ-opioid, σ1, and hERG; larger positive values indicate weaker mean predicted off-target binding relative to NMDA within this internal docking-based comparative framework.**

Identity note: R4/R16 and R5/R20 are retained as identifier-level rows from the submitted docking-source matrix. Because the submitted SMILES/SDF identity files indicate duplicate/alias structural entries for these pairs, these rows should not be interpreted as independent chemical scaffolds or as independent structure-activity evidence.
