# Supplementary material for: Structure-based molecular modeling of a novel S-Remiketamine scaffold and derived arylcyclohexanone analogues: NMDA receptor dynamics, MM-GBSA ranking, and CES1-Oriented structural compatibility
Source: Front Chem. 2026 Sep 3;14:1922077. doi: 10.3389/fchem.2026.1922077 (PMC13582596; doi:10.3389/fchem.2026.1922077)
Supplement: Supplementary file 3 [file DataSheet1.zip › Supplementary_Data_Sheet_2_Supplementary_Tables/Supplementary Table_S5_ADMET_metabolite_summary.docx]

**Supplementary Table S5. ADMET and predicted metabolite summary for prioritized compounds**

Table note. This supplementary table summarizes physicochemical descriptors, qualitative in silico ADMET interpretations, and explicitly recoverable predicted metabolite routes for the prioritized compounds R2, R11, and R14. These entries are predictive and hypothesis-generating, not experimental. Quantitative descriptor values are limited to values available in the final project source set. Qualitative ADMET and metabolite interpretations are restricted to conservative, manuscript-compatible statements and should not be read as experimental evidence of blood–brain barrier penetration, metabolic rate, enzymatic turnover, plasma clearance, metabolite formation, biological inactivity, or in vivo pharmacokinetics.

**Table S5A. Structured physicochemical descriptor summary**

| **Compound** | **Role** | **N-substituent** | **MW (Da)** | **Charge** | **Pred. pKa** | **TPSA (Å²)** | **LogP** | **HBD** | **HBA** | **Descriptor screen** |
| --- | --- | --- | --- | --- | --- | --- | --- | --- | --- | --- |
| R2 | Static docking/NMDA-preference benchmark | Isobutyryl | 323.1 | 0 | 7.7 | 56.0 | 2.8 | 1 | 4 | Within standard small-molecule medicinal-chemistry descriptor range |
| R11 | Mechanistic comparator | Glycolyl linker | 295.1 | 0 | 7.8 | 59.7 | 2.3 | 2 | 4 | Within standard small-molecule medicinal-chemistry descriptor range |
| R14 | Integrated computational candidate | Acetoacetyl | 321.1 | 0 | 7.6 | 59.4 | 2.4 | 1 | 4 | Within standard small-molecule medicinal-chemistry descriptor range |

**Table S5B. Submission-safe qualitative ADMET interpretation for prioritized compounds**

| **Compound** | **GI absorption** | **BBB / BOILED-Egg** | **P-gp** | **CYP liability summary** | **Solubility / permeability note** | **Submission-safe interpretation** |
| --- | --- | --- | --- | --- | --- | --- |
| R2 | High | Predicted BBB-permeant / CNS-oriented | Predicted non-substrate | No predicted CYP3A4/CYP2C9/CYP1A2 inhibition; possible CYP2C19 prediction flag noted | Balanced predicted permeability with lower calculated solubility than R11 | Supports a predicted CNS-compatible descriptor profile; relative metabolic persistence remains a hypothesis requiring experimental validation. |
| R11 | High | Reduced predicted BBB penetration; BOILED-Egg non-permeant in the source prediction output | Predicted non-substrate | No predicted CYP3A4/CYP2C9/CYP1A2 inhibition; comparatively fewer CYP prediction flags | Highest calculated relative solubility among prioritized compounds; any faster clearance remains predictive and requires experimental validation | Supports a more polar comparator profile; any faster clearance or reduced CNS residence remains predictive and requires experimental validation. |
| R14 | High | Predicted BBB-permeant / CNS-oriented | Predicted non-substrate | No predicted CYP3A4/CYP2C9/CYP1A2 inhibition; possible CYP2C19 prediction flag noted | Balanced predicted solubility and lipophilicity; compatible with the CNS-oriented design hypothesis and predicted downstream polar metabolism | Supports R14's integrated prioritization when interpreted alongside receptor docking, MD, MM-GBSA, CES1 active-site geometry, and off-target docking data. |

**Table S5C. Explicitly recoverable predicted metabolite routes**

| **Parent** | **Enzyme / system** | **Reaction type** | **Predicted metabolite formula** | **Interpretation** |
| --- | --- | --- | --- | --- |
| R14 | UGT (EC 2.4.1.17) | 1,3-Dicarbonyl C-glucuronidation | C22H26ClNO10 | Predicted polar glucuronide conjugate |
| R14 | CYP1A2, CYP2C8, CYP2C9, CYP3A4 | Hydroxylation of alicyclic carbon | C16H18ClNO5 | Predicted hydroxylated metabolite; functional activity not experimentally established |
| R14 | CYP2C19, CYP2D6 | Aromatic ring hydroxylation | C16H18ClNO5 | Predicted more polar phenolic derivative; functional activity not experimentally established |
| R14 | CYP2E1 | Para-hydroxylation on aryl chloride | C16H18ClNO5 | Predicted aromatic hydroxylation potentially enabling subsequent conjugation |
| R14 | CYP2A6, CYP2B6 | α-Hydroxylation of carbonyl group | C16H18ClNO5 | Predicted α-hydroxy-acetoacetyl derivative; downstream rearrangement or degradation remains hypothetical |
| R14 | Gut microbial oxidoreductases | Reduction of carbonyl to alcohol | C16H20ClNO4 | Predicted hydroxylated alcohol intermediate with increased polarity; activity and excretion route not experimentally established |
| R14 | Human carboxylesterase 1 (CES1) | Hydrolytic cleavage of acetoacetyl group | C16H18ClNO4 | Predicted hydroxymethyl Remiketamine-related derivative; experimental identity and functional activity not established |
| R14 | UGT1A9, UGT2B7 | Glucuronidation of hydroxyl group | C22H28ClNO10 | Predicted polar phase II conjugate; elimination route not experimentally established |

**Source basis and limitations.**

Physicochemical descriptor rows were compiled from the final project descriptor workbook. Qualitative ADMET summaries were restricted to statements explicitly recoverable from the final project source set and manuscript source records. Detailed predicted metabolite-route enumeration was most explicit for R14; therefore, R14 is presented as the worked metabolite-prediction example, while R2 and R11 metabolite routes are not over-interpreted. This supplementary table is intentionally conservative and should not be read as experimental pharmacokinetic, metabolic, or functional validation. Predicted metabolite formulae are retained as source-derived identifiers; formula-derived exact mass values are not inferred in this submission table.
