# Supplementary material for: Structure-based molecular modeling of a novel S-Remiketamine scaffold and derived arylcyclohexanone analogues: NMDA receptor dynamics, MM-GBSA ranking, and CES1-Oriented structural compatibility
Source: Front Chem. 2026 Sep 3;14:1922077. doi: 10.3389/fchem.2026.1922077 (PMC13582596; doi:10.3389/fchem.2026.1922077)
Supplement: Supplementary file 5 [file DataSheet6.zip › Supplementary_Data_Sheet_7_Reviewer2_Source_Trajectory_Structural_Addendum_FINAL_POLISHED.docx]

**Supplementary Data Sheet 7**

**Reviewer 2 Source-Trajectory and Structural-Methods Addendum**

*S-Remiketamine Frontiers in Chemistry manuscript ID 1922077*

**Scientific-use note:** This addendum was prepared specifically to answer Reviewer 2 without adding unsupported claims. RMSD/RMSF panels are reproduced from the uploaded Desmond/SID MD source reports. Qualitative charge maps are force-field/Gasteiger partial-charge visualizations generated from deposited identity structures and are not quantum-derived MEP surfaces. No experimental potency, hydrolysis, pharmacokinetic, efficacy or safety claim is made.

# 1. Reviewer 2 request-compliance matrix

| **Reviewer 2 request** | **Source-derived action now taken** | **Location** | **Compliance status** |
| --- | --- | --- | --- |
| R1-R20 explanation and improved molecular properties | Expanded R-series design/property explanation and table of descriptors/ligand efficiency. | Main manuscript Sections 2.2, 3.1, 3.2; Table S10 in this addendum. | Addressed explicitly |
| 3D structures of best candidates and improved Figure 1 | Added selected local-minimum conformers and qualitative charge visualizations for R2/R11/R14. | Revised Figure 1; Supplementary Figures S1-S2. | Addressed explicitly |
| Full set of 3D structures | Added full S-ketamine/S-Remiketamine/R1-R20 local-minimum 3D conformer projection panel. | Supplementary Figure S1. | Addressed explicitly |
| Protonation state according to pH | Added explicit conditional protonation-state statement; LigPrep/Epik logs document Epik use at pH 7.0, while MD/protein-preparation reports use near-physiological pH. | Main manuscript Section 2.2; Table S11. | Addressed explicitly |
| MEP maps and surface complementarity | Added qualitative partial-charge maps and source-derived contact-complementarity heatmaps; clearly labelled as non-quantum and non-binding-energy evidence. | Figure 1; Supplementary Figures S2 and S9; source_contact_complementarity_summary.csv; Methods/limitations. | Addressed explicitly |
| Docking details: site, box, scoring, search, flexibility | Expanded docking methods. CES1 source records include Ser221-centered grid and 20 x 20 x 20 A box; workflow treated as flexible-ligand/prepared-receptor Glide docking, not induced-fit. | Main manuscript Sections 2.3-2.4; Table S12. | Addressed explicitly |
| Why one NMDA structure | Added rationale for PDB 7EU8 as ketamine-relevant human NMDA template and acknowledged single-template limitation. | Main manuscript Sections 2.3, 4.5. | Addressed explicitly |
| Ligand efficiency | Added NMDA docking score/heavy-atom count ligand efficiency for the library and highlighted candidates. | Main manuscript Section 3.2; Table S10. | Addressed explicitly |
| Free-receptor MD | Clarified no matched free-receptor MD simulations were included; ligand-induced receptor changes are not claimed. | Main manuscript Sections 2.6, 4.5; Table S13. | Addressed explicitly |
| MM-GBSA frames | Added source-derived frame description: NMDA last 20 ns at 100 ps (~200 frames); CES1 reports 1000 stored frames and representative-frame/end-state reporting. | Main manuscript Section 2.6; Table S13. | Addressed explicitly |
| RMSD/RMSF figures | Added RMSD/RMSF plots from uploaded MD source reports for NMDA and CES1 complexes. | Supplementary Figures S7-S8. | Addressed explicitly |
| Protein region used for NMDA MD | Clarified prepared 7EU8 receptor-complex model without explicit membrane bilayer; not full physiological membrane-embedded NMDA channel modelling. | Main manuscript Sections 2.6, 4.5. | Addressed explicitly |
| Improve Figures 4 and 5 and add 2D diagrams | Added 2D NMDA interaction and CES1 active-site geometry summary diagrams. | Revised Figures 4-5; Supplementary Figures S3A-S3B. | Addressed explicitly |
| Limitations repeated | Consolidated limitation wording and used added space for method/result detail. | Main manuscript Discussion and Conclusions. | Addressed explicitly |

# 2. Table S10. R-series descriptor and ligand-efficiency summary

**Interpretation:** Ligand efficiency is docking score divided by heavy-atom count. It is an internal docking-normalization metric, not experimental ligand efficiency or binding affinity.

| **Compound** | **Formula** | **Heavy atoms** | **Exact MW** | **TPSA** | **cLogP** | **HBD** | **HBA** | **Formal charge** | **NMDA docking score** | **Docking LE** |
| --- | --- | --- | --- | --- | --- | --- | --- | --- | --- | --- |
| S-ketamine | C13H16ClNO | 16 | 237.092042 | 29.1 | 2.9 | 1 | 2 | 0 | -5.981 | -0.374 |
| S-Remiketamine | C15H18ClNO3 | 20 | 295.097521 | 55.4 | 2.79 | 1 | 4 | 0 | -6.448 | -0.322 |
| R1 | C15H19ClN2O3 | 21 | 310.10842 | 67.4 | 2.58 | 2 | 4 | 0 | -7.162 | -0.341 |
| R2 | C17H22ClNO3 | 22 | 323.128821 | 55.4 | 3.42 | 1 | 4 | 0 | -7.308 | -0.332 |
| R3 | C14H16ClNO3 | 19 | 281.081871 | 55.4 | 2.4 | 1 | 4 | 0 |  |  |
| R4 | C15H17Cl2NO3 | 21 | 329.058549 | 55.4 | 3.01 | 1 | 4 | 0 |  |  |
| R5 | C16H21ClN2O3 | 22 | 324.12407 | 67.4 | 2.97 | 2 | 4 | 0 | -6.949 | -0.316 |
| R6 | C17H22ClNO4 | 23 | 339.123736 | 64.6 | 3.79 | 1 | 5 | 0 |  |  |
| R7 | C13H16ClNO2 | 17 | 253.086956 | 49.3 | 2.22 | 2 | 3 | 0 |  |  |
| R8 | C17H20ClNO5 | 24 | 353.103 | 81.7 | 2.33 | 1 | 6 | 0 |  |  |
| R9 | C20H20ClNO3 | 25 | 357.113171 | 55.4 | 4.08 | 1 | 4 | 0 |  |  |
| R10 | C15H17Cl2NO2 | 20 | 313.063634 | 46.2 | 3.08 | 1 | 3 | 0 |  |  |
| R11 | C15H18ClNO3 | 20 | 295.097521 | 66.4 | 1.83 | 2 | 4 | 0 | -4.681 | -0.234 |
| R12 | C15H16ClNO3 | 20 | 293.081871 | 63.2 | 2.38 | 1 | 3 | 0 |  |  |
| R13 | C16H18ClNO4 | 22 | 323.092436 | 83.5 | 2.31 | 2 | 4 | 0 |  |  |
| R14 | C17H20ClNO3 | 22 | 321.113171 | 63.2 | 2.82 | 1 | 4 | 0 | -6.476 | -0.294 |
| R15 | C15H17ClN2O4 | 22 | 324.087685 | 95.5 | 2.06 | 3 | 3 | 0 |  |  |
| R16 | C15H17Cl2NO3 | 21 | 329.058549 | 55.4 | 3.01 | 1 | 4 | 0 |  |  |
| R17 | C17H21ClN2O5 | 25 | 368.113899 | 97.7 | 2.37 | 2 | 7 | 0 |  |  |
| R18 | C15H16ClNO4 | 21 | 309.076786 | 72.5 | 1.97 | 1 | 5 | 0 |  |  |
| R19 | C16H20ClNO2 | 20 | 293.118257 | 46.2 | 3.25 | 1 | 3 | 0 |  |  |
| R20 | C16H21ClN2O3 | 22 | 324.12407 | 67.4 | 2.97 | 2 | 4 | 0 | -6.949 | -0.316 |

# 3. Table S11. Protonation-state traceability

| **Source item** | **What it supports** | **Conservative wording used** |
| --- | --- | --- |
| LigPrep/Epik source logs | Native LigPrep log files in the uploaded source package document Epik invocation, including pH 7.0 microstate generation. | Supports explicit statement that protonation/microstate preparation was considered; does not justify claiming a complete experimental protonation ensemble. |
| Protein preparation/MD reports | Uploaded NMDA and CES1 MD reports state protein preparation with Epik/protonation states at near-physiological pH, including pH 7.4 or pH 7.4 +/- 0.5 language. | Supports near-physiological preparation wording. |
| Deposited SDF/SMILES files | SDF/SMILES identity files report formal charge 0 for the entries. | These are identity records and are not a full microstate ensemble; docking/MD interpretation is conditional on prepared states. |

# 4. Table S12. Docking-method traceability

| **Method item** | **Source-derived detail** | **Manuscript interpretation** |
| --- | --- | --- |
| NMDA receptor template | PDB 7EU8 | Ketamine-relevant human NMDA structural template used for internal comparability; single-template limitation acknowledged. |
| CES1 template | PDB 1MX9 | Human CES1 catalytic pocket model used for esterase-orientation assessment. |
| CES1 grid | Ser221-centered catalytic region; source record reports approximate centre -0.711080, -31.422480, -16.809360 and 20 x 20 x 20 A box. | Included in Methods as source-derived box/detail where available. |
| Scoring/workflow | Glide XP / Glide-style receptor-grid docking; flexible-ligand/prepared-receptor interpretation. | No induced-fit or flexible-residue docking claim is made unless supported. |
| CES1 geometry filters | Ser221 O-gamma-carbonyl C distance, oxyanion-hole contacts involving Gly142/Gly143/Ala222, His468/Glu354 catalytic environment. | Used only for structural compatibility, not hydrolysis-rate proof. |

# 5. Table S13. MD, RMSD/RMSF and MM-GBSA traceability

| **MD/MM-GBSA item** | **Source-derived detail** | **Use in revision** |
| --- | --- | --- |
| NMDA MD systems | R2, R11 and R14 receptor-ligand complexes using prepared PDB 7EU8 model. | 100 ns ligand-bound complex simulations; no matched free-receptor simulations in curated source set. |
| NMDA trajectory outputs | Uploaded MD report includes RMSD and RMSF plots for R2, R11 and R14. | Reproduced as Supplementary Figure S7. |
| NMDA MM-GBSA frames | Source method text states final/stabilized trajectory snapshots from the last 20 ns with 100 ps sampling; supporting text indicates about 200 frames. | Added to Methods as source-derived frame description. |
| CES1 MD systems | R2, R11 and R14 CES1-ligand complexes using PDB 1MX9 model. | 100 ns ligand-bound complex simulations with 1000 stored frames at 100 ps in source report. |
| CES1 trajectory outputs | Uploaded CES1 MD report includes RMSD and RMSF plots for R2, R11 and R14. | Reproduced as Supplementary Figure S8. |
| CES1 MM-GBSA frames | Source text reports representative frames every 10 ns and 0/100 ns reporting in the summary. | Interpreted as comparative MM-GBSA only, not experimental binding or catalytic evidence. |

# 6. Reviewer-requested structural and electrostatic visualizations

*Supplementary Figure S1. Local-minimum 3D conformer projections for S-ketamine, S-Remiketamine and R1-R20. Conformers were regenerated from deposited identity structures for visualization and are not experimental or receptor-bound conformations.*

*Supplementary Figure S2. Qualitative partial-charge maps for selected compounds. These are force-field/Gasteiger partial-charge visualizations, not quantum-derived MEP surfaces, and are used only for qualitative comparison of polar/non-polar regions.*

# 7. Reviewer-requested 2D interaction and geometry diagrams

*Supplementary Figure S3A. NMDA 2D interaction-summary diagram for R2, R11 and R14 based on tabulated interaction occupancy records.*

*Supplementary Figure S3B. CES1 2D active-site geometry summary for R2, R11 and R14 based on source geometric and occupancy descriptors.*

# 8. Reviewer-requested RMSD/RMSF source-report panels

**Traceability note:** The following RMSD/RMSF panels are reproduced from the uploaded MD simulation source reports. The underlying numeric time-series were not supplied as independent CSV files, so the figures are included as source-report panels rather than independently re-plotted curves.

*Supplementary Figure S7. NMDA receptor-ligand RMSD/RMSF panels for R2, R11 and R14 reproduced from the uploaded MD source report.*

*Supplementary Figure S8. CES1-ligand RMSD/RMSF panels for R2, R11 and R14 reproduced from the uploaded MD source report.*

# 9. Interpretation boundary

This addendum is intended to improve structural and methodological transparency in response to Reviewer 2. It does not convert the study into experimental pharmacology, enzymology, or PK/PD work. All figures and tables should be interpreted as computational, comparative and hypothesis-generating.

# 10. Final residual Reviewer 2 requests: electrostatic/surface complementarity and free-receptor MD audit

This final addendum section addresses the two residual reviewer-sensitive items explicitly and source-transparently. It uses only the uploaded/deposited source files and does not introduce unsupported simulations or claims.

## 10.1 Electrostatic-potential and binding-site complementarity visualization

Ligand electrostatic-potential-like surface maps were generated from the deposited SDF structure files using RDKit 3D coordinates and Gasteiger partial charges. For binding-site complementarity, the uploaded Desmond PL-Contacts files were parsed to calculate residue-level contact occupancy across available trajectory frames for polar/aromatic and hydrophobic contact classes in NMDA and CES1 ligand-bound complexes. These analyses provide qualitative structural/electrostatic visualization and source-derived contact-complementarity evidence. They are not quantum-mechanical MEP calculations and are not new binding-free-energy calculations.

**Supplementary Figure S9. Source-derived electrostatic and binding-site complementarity analysis.** Panel A shows ligand electrostatic-potential-like surface maps for S-ketamine, S-Remiketamine, R2, R11 and R14 generated from deposited SDF structures using RDKit 3D coordinates and Gasteiger partial charges. Panels B-E show source-derived binding-site contact-complementarity heatmaps calculated from uploaded Desmond PL-Contacts files as residue contact occupancy (% of frames) for ligand-bound NMDA and CES1 complexes. These maps support qualitative comparison of polar/non-polar ligand regions and their source-observed contact environments, without implying quantum-derived MEP or quantitative surface-energy calculations.

The source-derived contact-complementarity summary CSV is included with this addendum as source_contact_complementarity_summary.csv.

## 10.2 Free-receptor MD source audit

Reviewer 2 asked whether free receptors were simulated. A direct audit of the uploaded source files was performed. The source package contains ligand-bound NMDA and CES1 complex MD reports, trajectory-analysis reports, PL-contact files and time-point trajectory images for R2, R11 and R14. No matched apo/free-receptor production MD report, trajectory, RMSD/RMSF time-series, PL-contact file, or time-point image series was identified. Therefore, the study is explicitly interpreted as a comparative ligand-bound complex analysis, not as a ligand-induced conformational-change study relative to an apo receptor control.

| **Source item audited** | **Files reviewed** | **Finding for free-receptor / apo MD** |
| --- | --- | --- |
| MD simulation reports | 4-MD Simulation Reports/MD Simulations Studies of Compounds on NMDA Receptors.docx; 4-MD Simulation Reports/MD Simulation Studies of Compound on Esterase Enzyme.docx | Reports describe ligand-bound receptor/enzyme complexes for R2, R11 and R14; no matched apo/free-receptor production simulation report identified. |
| Trajectory analysis reports | 5-Trajectory Analysis/Trajectory Analysis For R2,R11,R14 with NMDA.docx; 5-Trajectory Analysis/Trajectory Analysis For R2,R11,R14 with Esterase.docx | Trajectory reports contain ligand-bound complex analyses and frames for R2, R11 and R14; no standalone apo/free-receptor trajectory report identified. |
| Trajectory image folders | 2. Bond Interaction Data/Trajectory images/Trajectory analysis/*.png | All available time-point images are labelled by ligand-complex systems (R2/R11/R14 with NMDA or esterase); no apo/free-receptor time-point series identified. |
| Quantitative and contact files | 2. Bond Interaction Data PL-Contacts files; 6-Quantitative Analyses; 7-Supporting Information | Contact, MM-GBSA and geometry files are ligand-bound analyses; no apo/free-receptor contact, RMSD/RMSF or MM-GBSA dataset identified. |
| Manuscript interpretation | Revised manuscript and response documents | The study now explicitly states that matched free-receptor MD was not included and that ligand-induced receptor conformational change relative to apo receptor is not inferred. |

Supplementary Table S14. Source audit for the reviewer-requested free-receptor MD clarification.
